# Transcriptional Activation of the Adenoviral Genome Is Mediated by Capsid Protein VI

**DOI:** 10.1371/journal.ppat.1002549

**Published:** 2012-02-23

**Authors:** Sabrina Schreiner, Ruben Martinez, Peter Groitl, Fabienne Rayne, Remi Vaillant, Peter Wimmer, Guillaume Bossis, Thomas Sternsdorf, Lisa Marcinowski, Zsolt Ruzsics, Thomas Dobner, Harald Wodrich

**Affiliations:** 1 Heinrich–Pette–Institute, Leibniz Institute for Experimental Virology, Hamburg, Germany; 2 Microbiologie Fondamentale et Pathogénicité, MFP CNRS UMR 5234, Université Bordeaux SEGALEN, Bordeaux, France; 3 Institut Génétique Moléculaire de Montpellier, Université Montpellier 1, Université Montpellier 2, IGMM CNRS UMR 5535, Montpellier, France; 4 Research Institute, Children's Cancer Center, Hamburg, Germany; 5 Gene Center Munich, Ludwig-Maximilian University, Munich, Munich, Germany; University of Michigan, United States of America

## Abstract

Gene expression of DNA viruses requires nuclear import of the viral genome. Human Adenoviruses (Ads), like most DNA viruses, encode factors within early transcription units promoting their own gene expression and counteracting cellular antiviral defense mechanisms. The cellular transcriptional repressor Daxx prevents viral gene expression through the assembly of repressive chromatin remodeling complexes targeting incoming viral genomes. However, it has remained unclear how initial transcriptional activation of the adenoviral genome is achieved. Here we show that Daxx mediated repression of the immediate early Ad E1A promoter is efficiently counteracted by the capsid protein VI. This requires a conserved PPxY motif in protein VI. Capsid proteins from other DNA viruses were also shown to activate the Ad E1A promoter independent of Ad gene expression and support virus replication. Our results show how Ad entry is connected to transcriptional activation of their genome in the nucleus. Our data further suggest a common principle for genome activation of DNA viruses by counteracting Daxx related repressive mechanisms through virion proteins.

## Author Summary

To initiate infection, DNA viruses deliver their genome to the nucleus and express viral genes required for genome replication. Efficient transport is achieved by packing the viral genome as a condensed, transcriptionally inactive nucleo-protein complex. However, for most DNA viruses, including Adenoviruses (Ads), it remains unclear how the viral genome is decondensed and how transcription is initiated inside the nucleus. Cells control unwanted gene expression by chromatin modification mediated through transcriptionally repressive complexes. A key factor in repressive complex assemblies is the transcriptional repressor Daxx. The Ad structural capsid protein VI is required for endosomal escape and nuclear transport. Here we show that protein VI also activates the Ad E1A promoter to initiate Ad gene expression. This is achieved through the removal of Daxx repression from the E1A promoter, which requires a conserved ubiquitin ligase interacting motif (PPxY-motif) in protein VI. We further show that capsid proteins from other unrelated DNA viruses also activate the Ad E1A promoter and support Ad replication by counteracting Daxx repression, functionally replacing protein VI. Our data suggest that reversal of Daxx repression by virion proteins is a widespread mechanism among DNA viruses that is not restricted to a single virus family.

## Introduction

DNA viruses require the transport of their genome into the nucleus to initiate replication. Cells perceive the introduction of foreign nucleic acids or unscheduled replication as danger signals and activate a DNA damage response that leads to cell cycle arrest and/or apoptosis. To ensure proper replication, DNA viruses express ‘early’ viral genes to degrade or displace key regulators of cellular antiviral machinery. In return, cells repress incoming viral genomes through a network of transcriptional repressors and activators that normally control cellular homeostasis [reviewed in 1], [2].

The nuclear domains thought to be responsible for repressing viral genomes are ND10 or promyelocytic nuclear bodies [PML]-[NBs; reviewed in 3,4] named after the scaffolding PML protein. PML-NBs are interferon inducible, dot-like nuclear structures associated with proteins with transcriptional repressive functions. These include HP-1, Sp100, ATRX and Daxx [summarized in 4], [5]. Daxx (death domain associated protein) was first described as a modulator of Fas-induced apoptotic signaling [6]. When chromatin-bound, Daxx inhibits basal gene expression from various promoters by binding to transcription factors (e.g. p53/p73, NF-kappaB, E2F1, Pax3, Smad4 or ETS1), ATRX, histone deacetylases and core histones to form a repressive chromatin environment [7]–[13]. In contrast, Daxx localization to PML-NBs reduces its repressive capacity and facilitates apoptosis through p53 family members [5], [7], [14].

PML-NBs are found in close proximity to replication centers of DNA viruses (e.g. adenoviruses (Ads), herpes simplex virus (HSV-1), human cytomegalovirus (HCMV) and human papillomavirus [HPV]; [ 15], [16]–[18]. Gene expression from these viruses is repressed via the PML-NBs, suggesting a role in antiviral defense [19]–[22].

To counteract genome repression, viral genome activation involves PML-NB disruption or degradation of Daxx, Sp100 and/or PML via different mechanisms. HCMV gene expression is initiated by proteasomal degradation of Daxx via tegument protein pp71 of the incoming particle [23]. Early HSV-1 gene expression requires PML degradation, mediated by the virus encoded ubiquitin ligase ICP0. Furthermore, in order to activate viral gene expression, transcriptional repression by Daxx and ATRX needs to be relieved [3], [24], [25]. HPV early gene expression is supported by reorganization of PML-NBs through the minor capsid protein L2 [26].

At the beginning of infection, Ads express the immediate early protein E1A from the E1A promoter. E1A binds and displaces the transcriptional repressor Rb from E2F transcription factors. This results in the auto-stimulation of E1A expression and the activation of the downstream viral expression units E1B, E2, E3 and E4 as well as promoting cellular gene expression. The early E1B-55K protein forms a SCF-like E3-ubiquitin ligase complex with the viral E4orf6 and several cellular factors. This complex degrades factors (for example, factors of the DNA damage response) to ensure progression of the replication cycle [summarized in 1], [2], [27]. E1B-55K protein complex also targets Daxx for proteasomal degradation counteracting its repressive effect [21]. In contrast to HSV-1, PML is not degraded by Ads but relocalized into track-like structures through the E4orf3 protein [28], [29].

Despite the well-characterized mechanism of E1A dependent transactivation of early Ad genes, it is unclear how the E1A transcription is efficiently initiated before other viral genes are expressed. The genome enters the cell as a transcriptionally inactive nucleoprotein complex, which is highly condensed by the histone-like viral protein VII inside the capsid shell. Partial disassembly of the endocytosed capsid releases the endosomolytic internal capsid protein VI, permitting endosomal membrane penetration [30], [31] and transport towards the nucleus. After import through the nuclear pore complex, Ad genomes associate with PML-NBs and replication centers are established [30], [31], [reviewed in 32], [33]–[35]. Endosomal escape and subsequent transport are facilitated by Nedd4 ubiquitin ligases, which are recruited through a conserved PPxY motif in protein VI. Ads with mutated PPxY motif do not bind Nedd4 ligases and have reduced infectivity, showing the importance of this interaction for the onset of gene expression from the viral genome [36].

Here we report that Ad capsid proteins and cytoplasmic entry steps are linked to initiation of the adenoviral E1A expression by counteracting Daxx mediated transcriptional repression. Using the Ad system, we further show that capsid proteins from several other DNA viruses share and complement this function. This suggests a conserved mechanism among DNA viruses and provides insights into the very early virus-host interactions required to establish an optimal cellular environment for productive infection.

## Results

### Ad with PPxY-mutated protein VI exhibits reduced replication fitness

The capsid protein VI participates in two crucial steps in the nuclear delivery of the Ad genome. Firstly, protein VI is required for lysis of endosomal membranes. Secondly, it is needed for efficient post-endosomolytic transport, mediated by the cellular ubiquitin ligase Nedd4 that binds to a conserved PPxY motif in protein VI. Mutating the PPxY motif interferes with capsid transport toward the nucleus and efficient viral gene expression [30], [36].

**Figure 1:**
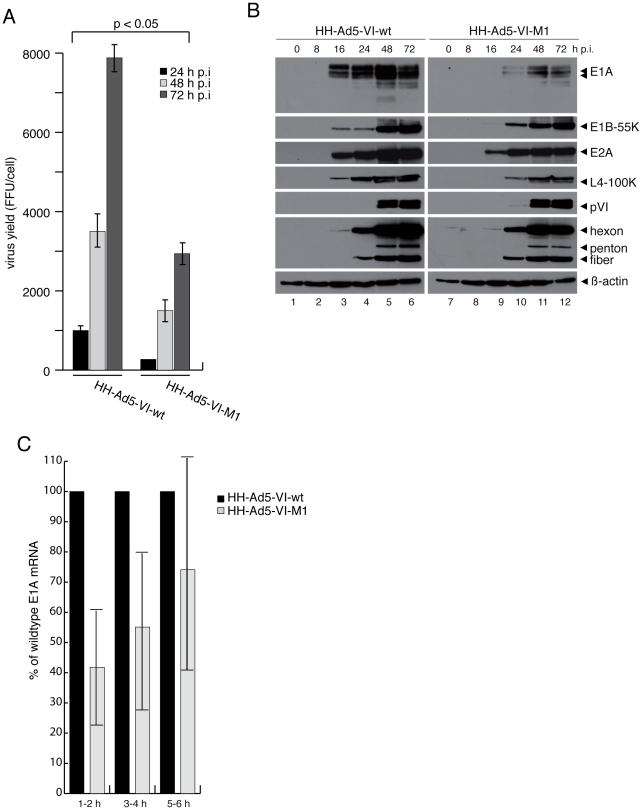
Viruses with mutated PPxY motif in protein VI (M1) have altered gene expression. (A) U2OS cells were infected with replication competent HH-Ad5-VI-wt or HH-Ad5-VI-M1 with a multiplicity of infection (MOI) of 50 FFU/cell (see Figure S1). Viral particles were harvested at 24, 48 and 72 h p.i. and virus yield was determined by quantitative E2A stain. The results represent the average from three independent experiments. (B) U2OS cells were infected with HH-Ad5-VI-wt or HH-Ad5-VI-M1 at a MOI of 50 FFU/cell and whole-cell extracts were prepared after indicated time-points and subjected to immunoblotting (IB). Corresponding proteins are indicated to the right. MOI dependent replication differences are shown in Figure S2. (C) Cells were infected as in A and newly synthesized RNAs were labelled for the time p.i. as indicated on the x-axis and described in the materials and methods section. Extracted RNAs were reverse transcribed and quantified using qPCR using exon-spanning E1A specific primers and normalized against GAPDH mRNA levels. E1A mRNA levels in wt-infected cells were arbitrarily set to 100%. Data are derived from three independent experiments.

To investigate the role of protein VI during post-endosomolytic steps required for the onset of viral replication, we constructed replication competent Ads containing the E1 region with either wildtype (wt) protein VI (HH-Ad5-VI-wt, depicted in the Figure S1) or mutant “M1” protein VI in which the PPSY motif was mutated to PGAA that abolished Nedd4 interaction [HH-Ad5-M1; Fig. S1; [36]]. Following infection of U2OS cells, we observed that M1 virus replication was attenuated compared to wt (Figure 1A and S1B). This is in agreement with our previous observations showing reduced infectivity of an E1-deleted M1 Ad vector compared to the corresponding E1-deleted wt Ad vector [36]. To distinguish between capsid transport and possible more downstream effects, we infected cells with different amounts of replication competent wt and M1 viruses. Then, we determined the genome copy numbers in nuclear and cytoplasmic fractions by qPCR and the efficiency of the initiation of virus replication by quantification of E2A stained replication centers (detailed in Figure S2). Compared to wt, fewer M1 virus genomes accumulated in the nucleus associated fraction, independent of the amount of input virus. In contrast, initiation of virus replication for M1 genomes was reduced for low, but not at high physical particle per cell ratios (Figure S2) suggesting defects downstream of virus nuclear transport.

Therefore, the expression of the early viral proteins E1A, E1B-55K and E2A in wt and M1 infected cells was analyzed by western blot, starting 8 h post infection (p.i.) and throughout the whole replication cycle (Figure 1B, left panel). We observed that expression of E1A in M1 virus infected cells was reduced compared to wt (Figure 1B, right panel) and accordingly, all other gene products were expressed with a delayed kinetic. This observation can be explained by the initial lower levels of E1A expression, because E1A is required for full activity of Ad downstream promoters [37]. Thus, we next investigated if the reduced E1A protein expression in M1-infected cells was due to reduced transcriptional activation of the E1A promoter following infection. We isolated and quantified newly synthesized E1A mRNA from cells infected with wt and M1 virus starting as early as 1–2 h p.i. (Figure 1C). The results confirmed that, at 1–4 h p.i., M1-infected cells showed reduced levels of newly synthesized E1A mRNA compared to wt-infected cells. Interestingly this reduction was gradually compensated throughout the first hours of infection (Figure 1C, compare 1–2 h, 3–4 h and 5–6 h) suggesting that low levels of initially made E1A were sufficient to compensate for the M1-defect in E1A transcription.

The high particle per cell ratio requirement for transcriptional activation and the reduced levels of E1A mRNA and E1A protein expression for the M1 virus indicated that the PPxY motif in protein VI not only affects transport towards the nucleus, but also early viral gene expression, presumably through separate mechanisms.

### Capsid protein VI of incoming Ads is targeted to PML-NBs

**Figure 2:**
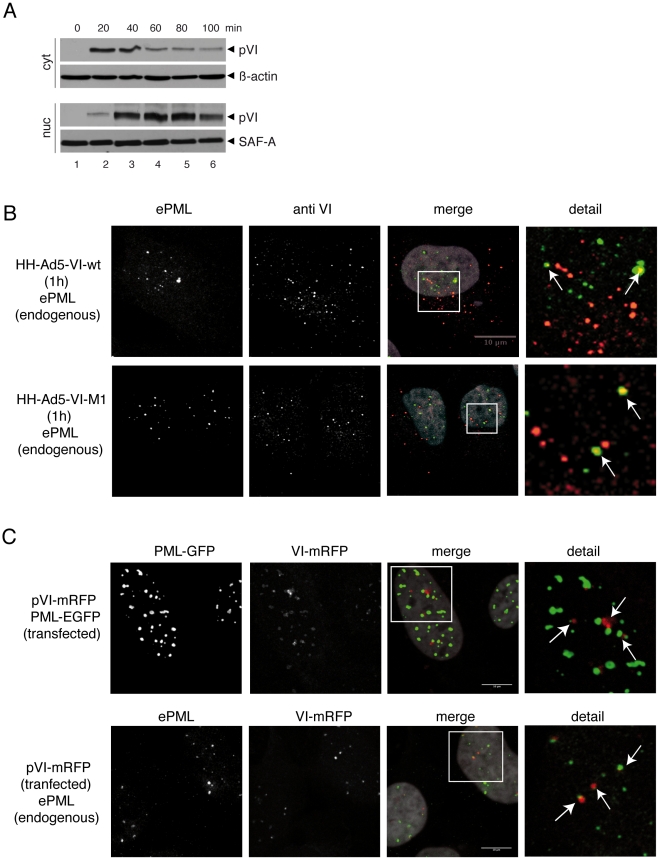
Nuclear accumulation of protein VI at PML-NBs during entry. (A) U2OS cells were infected with HH-Ad5-VI-wt at a MOI of 1000 FFU/cell and fractionated at 20 min intervals and subjected to IB using serum against protein VI, polyclonal Ab (pAb) against the splicing factor SAF-A (nuclear fraction) and monoclonal Ab (MAb) against β-actin (cytoplasmic fraction) as indicated to the right. (B) U2OS cells were synchronously infected with HH-Ad5-VI-wt (top row) or HH-Ad5-VI-M1 (bottom row) and fixed after 1 h. Endogenous PML (first column) and virus, derived protein VI (second column), were detected with specific Ab. In the overlay PML is shown in green and protein VI in red (third column). A detailed magnification of the white rectangle is given in the fourth column. (C) U2OS cells were transfected with mRFP-tagged protein VI and GFP-tagged PML expression vectors (top row) or mRFP-tagged protein VI followed by Ab stain of ePML (bottom row). Intracellular localization of PML (first column), protein VI (second column) or an overlay of both is shown (colors as above, third column). Colocalization is indicated by arrows (fourth column) and occurred in all transfected cells. A mapping of the interaction between VI and PML is shown in Figure S3.

We previously showed that protein VI contains nucleo-cytoplasmic transport signals [38]. To test if protein VI could play a direct role in the initial activation of the viral genome, we first analyzed whether protein VI from incoming Ad capsids is imported into the nucleus. Using nucleo-cytoplasmic fractionation, we observed rapid protein VI accumulation in the nuclear fraction after infection (Figure 2A).

Fractionation does not discriminate between nuclear (inside) or nucleus-associated (outside) accumulation of protein VI (e.g. capsid-associated at the microtubule organizing center). Thus, we investigated the subcellular localization of protein VI derived from entering viral particles by confocal microscopy in synchronous infected cells. Within one hour, we observed protein VI specific signals in dot-like structures inside the nucleus for wt- and the M1-virus. Using antibodies (Ab) against PML, we showed some protein VI associated with PML-NBs (Figure 2B).

We confirmed the association of some protein VI with PML-NBs in a virus free system by transfecting protein VI-mRFP alone or together with EGFP-PML expressing plasmids into U2OS cells. Transfected proteins were detected via the mRFP and EGFP signal or with specific Ab for endogenous PML (“endogenous” highlighted throughout the text and in figures by the suffix “e”, e.g. ePML). The results show that protein VI was able to independently associate with PML-NBs (Figure 2C). Using a serie of protein VI mutants, we mapped the region of protein VI required for PML-NB association (Figure S3). This analysis revealed that the N-terminal amphipathic helix was required for efficient PML-NB targeting, because a mutant (VI-delta54) deleted of the amphipathic helix showed a diffuse nuclear distribution (Figure S3). We repeatedly observed the clustering of PML in transfected cells, suggesting PML-NB structure modulation resulting from protein VI expression. In summary, these data showed that some protein VI from incoming Ad particles is targeted into the nucleus, where some of it consistently localizes adjacent to PML-NBs, suggesting an involvement in additional intranuclear steps.

### Protein VI interacts with and counteracts the PML-NB associated factor Daxx

**Figure 3:**
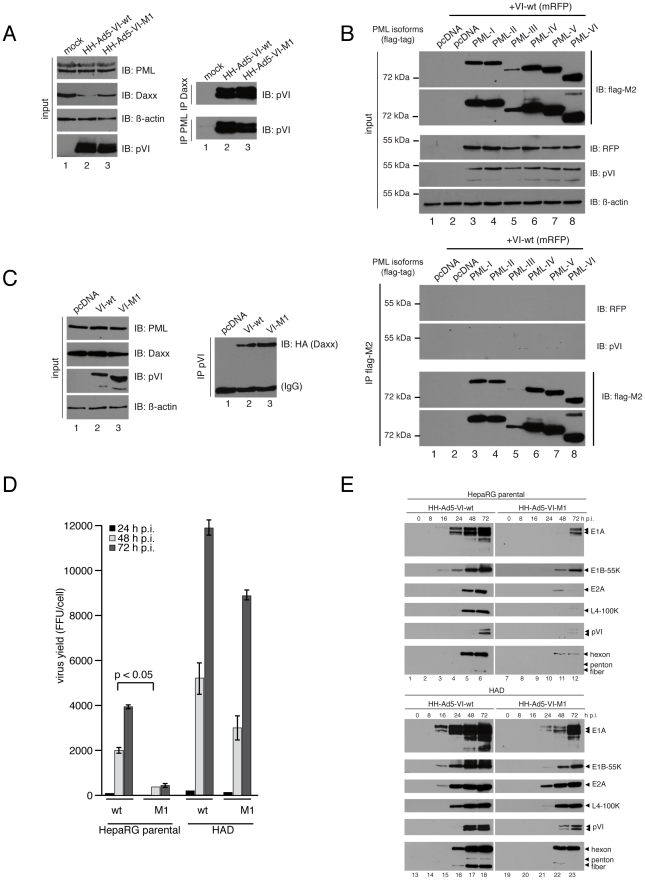
Protein VI interaction with PML and the PML-NB associated factor Daxx and rescue of HH-Ad5-VI-M1 replication by Daxx depletion. (A) H1299 cells were infected with HH-Ad5-VI-wt or HH-Ad5-VI-M1 at a MOI of 50 FFU/cell. Total-cell extracts were prepared 36 h p.i. and subjected to IB using specific Ab as indicated. Right panel : co-immunoprecipitation (IP) of protein VI was performed with Daxx-/PML-specific Ab followed by IB and detection of co-precipitated protein VI. (B) H1299 cells were transfected with mRFP tagged VI-wt and different constructs encoding for N-terminal flag-tagged human PML-isoforms I-VI and harvested after 24 h. Total-cell extracts were subjected to IB using Ab against flag-tag, RFP, VI or β-actin (top panel). IP of PML-isoforms was done using flag-specific MAbs M2. Detection of co-precipitated protein VI was done with serum against pVI or MAb against RFP. Immunoprecipitated PML proteins were detected with flag-specific MAb M2 (bottom panel). (C) H1299 cells were transfected with HA-tagged Daxx, RFP-tagged VI-wt and VI-M1 proteins. Total-cell extracts were prepared 24 h p.i. and analyzed by IB using specific Ab shown to the right. IP of protein VI was done with serum against pVI. Note that the size difference between VI-wt and VI-M1 results from fusion to mRFP (wt) or mCherry (M1). (D) HepaRG parental and HAD cells were infected with HH-Ad5-VI-wt or HH-Ad5-VI-M1 at a MOI of 50 FFU/cell. Viral particles were harvested at 24, 48 and 72 h p.i. and virus yield was determined by quantitative E2A stain. The results represent the average from three independent experiments (+/− STD). (E) HepaRG parental (top) and HAD (bottom) cells were infected with HH-Ad5-VI-wt or HH-Ad5-VI-M1 at a MOI of 50 FFU/cell and whole-cell extracts were prepared after indicated time-points. Proteins were subjected to IB using Ab specific for viral proteins as indicated on the right.

It was recently reported by some of the co-authors of this work that the transient PML-NBs resident factor Daxx suppressed Ad replication and was degraded late in the infection cycle [21]. The observation that some protein VI was associated with PML-NBs prompted us to investigate whether PML itself, or PML-NB-associated factors such as Daxx, interact with protein VI. These interactions could provide an explanation for the reduced transcription of the E1A promoter observed for the M1 virus. Cells were infected with HH-Ad5-VI-wt or -VI-M1 and harvested after 24 h. Lysates were subjected to immunoprecipitation (IP) using PML or Daxx specific Ab and analyzed by western blot (Figure 3A). The data showed that protein VI could be precipitated from both wt and M1 infected cells using either PML or Daxx specific Ab. In contrast to virus infected cells, we did not detect co-precipitated protein VI following cotransfection and IP with different PML isoforms, suggesting an indirect association of PML and protein VI, presumably bridged by other viral or infection induced factors (Figure 3B). In contrast, co-IP of protein VI with Daxx also occurred after isolated transfection of protein VI-wt as well as protein VI-M1 suggesting that the interaction is independent of other viral factors (Figure 3C). We next asked whether Daxx interaction with protein VI could explain the reduced replication of HH-Ad5-VI-M1. For these assays, we used the hepatoma derived cell line HepaRG, because of its close resemblance to primary cells [39], and HepaRG cells depleted of Daxx (HAD, Daxx was depleted with shRNA expressing lentiviral vectors [20]). We infected Daxx-depleted HAD and HepaRG parental cells with HH-Ad5-VI-wt and HH-Ad5-VI-M1 and determined virus yields and gene expression at 12, 24 and 72 h p.i. (Figure 3). The M1 virus was more strongly attenuated in HepaRG cells than in U2OS cells (compare to Figure 1), while Daxx depletion strongly enhanced virus production for both viruses and nearly restored the M1 virus yields to wt levels (Figure 3D). This improvement of Ad permissivity was confirmed by an increase of expression of all analyzed viral genes, including gene products from the E1A and E1B promoters (Figure 3E).

The data showed that Daxx depletion was sufficient to increase Ad gene expression for both viruses, emphasizing the role of Daxx in viral genome repression. In addition, wt but not M1 mutant protein VI could counteract Daxx mediated inhibition indicating that the PPxY motif of protein VI plays a significant role in initiating viral gene expression.

### PPxY motif is essential to reverse Daxx-mediated repression of Ad E1 promoters

**Figure 4:**
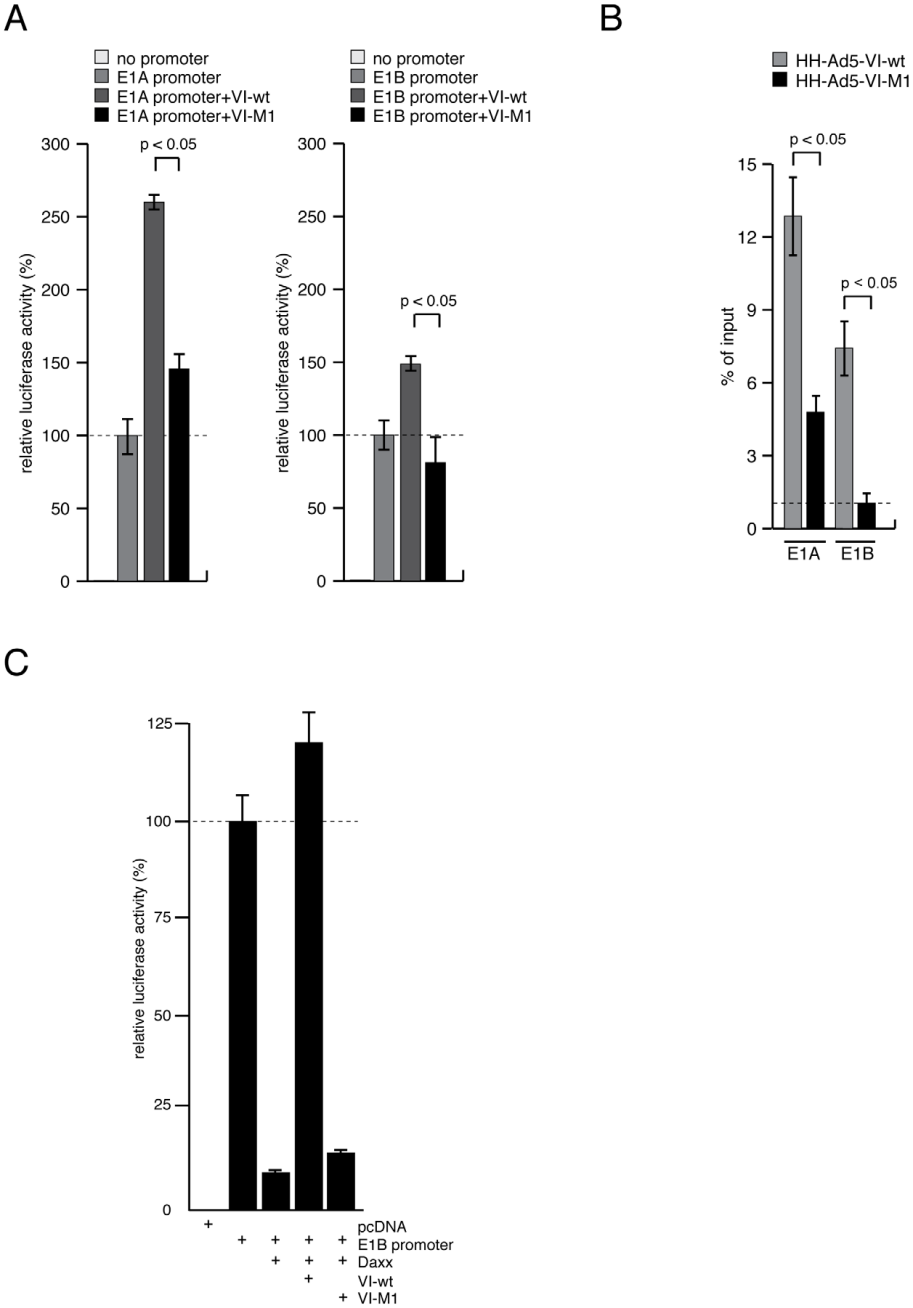
Protein VI mediates Ad transcriptional activation. (A) H1299 cells were transfected with luciferase reporter plasmids under E1A promoter- (left panel), E1B promoter- (right panel) or promoterless control and effector plasmids expressing VI-wt or VI-M1. Forty eight hours after transfection, samples were lysed, absolute luciferase activity was measured and activity of the E1 promoter alone was normalized to 100%. Mean and STD are from three independent experiments. Effects on additional viral promoters are shown in Figure S4. (B) H1299 cells were infected with HH-Ad5-VI-wt or HH-Ad5-VI-M1 at a MOI of 50 FFU/cell. Forty eight hours p.i., cells were fixed with formaldehyde and ChIP analysis was performed as described in materials and methods. The average C_t_-value was determined from triplicate reactions and normalized with standard curves for each primer pair. The y-axis indicates the percentage of immunoprecipitated signal from the input ( = 100%). (C) HAD cells were transfected with E1B promoter constructs and effector plasmids encoding for Daxx, VI-wt, VI-M1. Forty eight hours after transfection, samples were lysed and analyzed as in (A). Mean and STD are from three independent experiments.

Next, we asked whether the Ad immediate early E1A and early E1B promoters are targeted by Daxx mediated repression and if this is the case whether it can be reversed by protein VI. To this end, we constructed luciferase expression vectors controlled by the Ad E1A and E1B promoters and measured luciferase expression in protein VI-wt or protein VI-M1 transfected H1299 cells (Figure 4A). Unlike VI-M1, VI-wt was able to stimulate expression from the E1A promoter ∼2.5-fold and ∼1.5-fold from the E1B promoter (Figure 4A). To show direct association of protein VI with E1 promoters, we performed chromatin immunoprecipitation assays (ChIP) at 48 h p.i from M1- or wt virus infected cells, using protein VI specific serum and Ad promoter-specific primers (Figure 4B). The results show that the VI-wt protein was much more strongly associated with the E1A and E1B promoter in infected cells than the VI-M1 protein, which is also reflected in their relative activation ability (Figure 4B, compare with 4A). To analyze whether protein VI associated activation of Ad early promoters is involved in Daxx de-repression, we cotransfected the E1B promoter driven luciferase expression vector in absence or presence of Daxx with protein VI-wt or VI-M1 expression vectors. Protein VI-wt, but not VI-M1, alleviated Daxx repression implying a role for the PPxY motif (Figure 4C). Although there was less binding to protein VI compared to the E1A promoter, we observed a strong effect on the activation of luciferase expression in that experiment. We also tested if protein VI (wt or M1) stimulates other Ad promoters using luciferase expression vectors for all viral promoters. The data showed that protein VI-wt was able to stimulate most of the Ad promoters in absence of other viral factors to various degrees (Figure S4). The strongest induction was observed for the immediate early E1A and E2A early promoter, which is in agreement with the weak E2A expression observed in HepaRG cells in M1-virus infected cells and the restoration of E2A expression following Daxx depletion (see Figure 3E). In contrast, E3 and E4 promoter activation was weak with no clear difference between wt and M1. In the context of an ongoing virus infection, the transcriptional activation of both promoter groups (E1/E2 vs. E3/E4) was shown to be regulated by E1A but via different mechanisms [40], [41]. Thus, our data showed that protein VI might also play a minor role in the transcriptional activation of the E1/E2 promoter group.

Altogether, the promoter analysis suggests that protein VI plays a so far not recognized role in the Ad gene expression program.

### Daxx is translocated into the cytoplasm by protein VI

We next asked how the PPxY motif of protein VI contributes to Daxx de-repression. In previous work, we showed that this motif mediates protein VI interaction with cytoplasmic Nedd4 ubiquitin ligases [36]. Overexpression of protein VI and/or Nedd4 did not result in a change of steady-state Daxx levels (data not shown) suggesting that de-repression was not achieved through Daxx degradation as e.g. as shown for HCMV. However, when we tested if protein VI targets Nedd4 ligases to PML-NBs our analysis showed that protein VI-wt, but not VI-M1 targets Nedd4 ligases towards PML-NBs. This targeting required the PPxY motif and the amphipathic helix, but was independent of catalytical Nedd4 activity suggesting that Nedd4 ligases could be involved in other steps of counteracting Daxx repression by protein VI (Figure S5).

**Figure 5:**
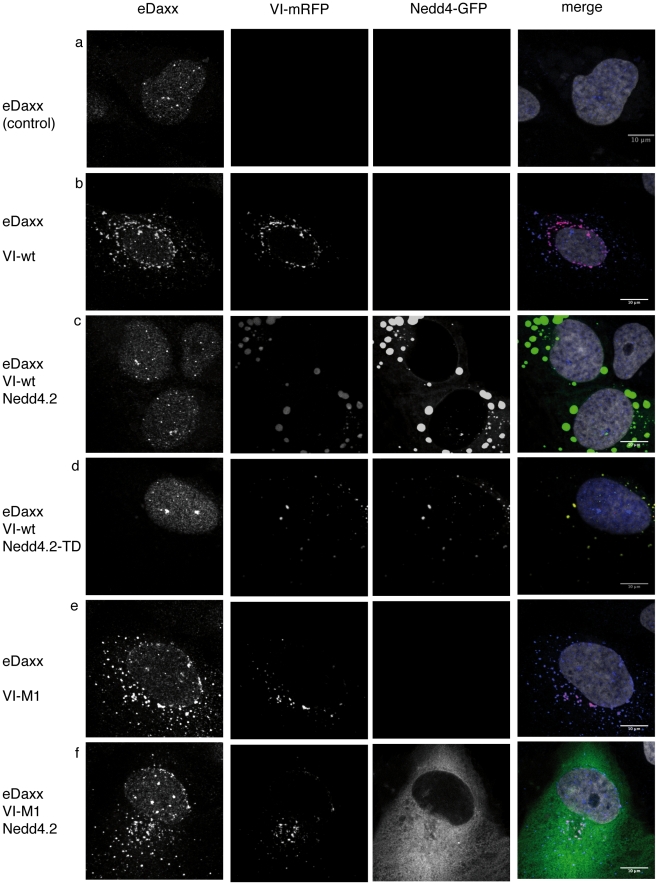
Nedd4 binding to the PPxY prevents cytoplasmic translocation of Daxx by protein VI. Endogenous Daxx in U2OS cells was detected following Mock transfection (A), transfection with VI-wt (B) or after cotransfection of VI-wt with Nedd 4.2 (C) or catalytical inactive Nedd 4.2-TD (D), transfection of VI-M1 (E) or VI-M1 cotransfected with Nedd 4.2. (F) as indicated to the left of each row. Daxx was stained with Ab against the endogenous protein (first column), VI was detected using the RFP signal (second column) or GFP for Nedd4 (third column). An overlay is shown in the last column showing Daxx in blue, protein VI in red, Nedd4 in green and the nucleus in grey. Phenotypes shown in representative cells were observed in all transfected cells. Colocalization of VI, Nedd4.2 and PML is shown in Figure S5.

As a next step, we therefore analyzed whether the subcellular distribution of Daxx was altered in response to protein VI and Nedd4 expression. In non-transfected cells, endogenous Daxx (eDaxx) is nuclear in steady state with some Daxx localizing to dot-like intranuclear structures resembling PML-NBs (Figure 5a). When we transfected expression vectors for protein VI-wt or VI-M1 into U2OS cells, nuclear localization of eDaxx was lost and eDaxx colocalized with transfected protein VI in the cytoplasm (Figure 5b and e). In contrast, following transfection of expression vectors for protein VI-wt and Nedd4 ligases, eDaxx remained nuclear and instead protein VI-wt colocalized with Nedd4 ligases in the cytoplasm (Figure 5c). When we transfected expression vectors for Nedd4 ligases and protein VI-M1, protein VI retained the capacity of translocating eDaxx to the cytoplasm (Figure 5f). These data suggested that binding of Nedd4 to the PPxY motif of protein VI efficiently competed with protein VI-dependent cytoplasmic translocation and/or cytoplasmic retention of Daxx. This effect did not require Nedd4 ubiquitin ligase activity (Figure 5d). Thus, our results suggested that the PPxY motif present in wt protein VI could influence the dynamic nucleo-cytoplasmic distribution of Daxx.

### Protein VI displaces Daxx from PML-NB

**Figure 6:**
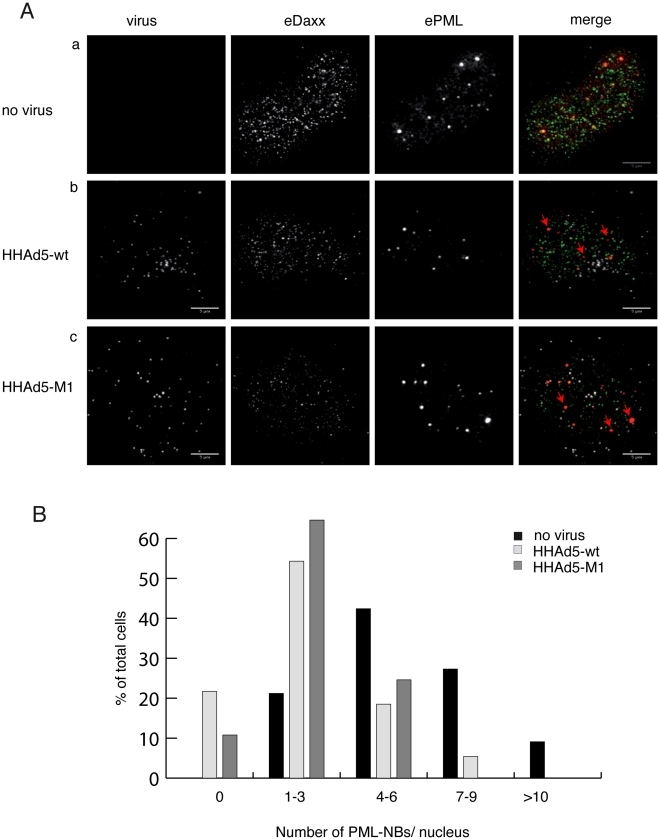
Daxx is displaced from PML bodies following Ad entry. (A) Endogenous Daxx (second column) and PML (third column) localization was determined in U2OS (a–c) cells either in the absence of infection (a) or 1 h after a synchronous infection using 100 physical particles of Alexa647 labeled HH-Ad5-VI-wt (b) or HH-Ad5-VI-M1 (c) per cell (first column). In the overlay (last column) virus is depicted in white, Daxx in green and PML in red. Red arrows indicate PML without Daxx colocalization. Daxx-PML colocalizations following transfection of VI is shown in Figure S7. (B) PML-NBs were counted in the nucleus at 1 h p.i. of non-infected cells (black bars) and cells infected as above with HHAd5-VI-wt (light grey bars) and HHAd5-VI-M1 (dark grey bars) classed into groups as indicated on the x-axis. Over 60 cells were counted per condition. Significant difference between non-infected vs. wt and non-infected vs. M1 infected cells was calculated using two-tailed 2-sample T-test (p<0.001).

To continue our analysis in a more physiological setting, we analyzed the subcellular localization of Daxx during Ad entry (Figure 6). In uninfected control cells, Daxx localized to the nucleoplasm and into PML-NBs. Within the first hour of infection, Daxx remained largely nuclear in wt- as well as M1-virus infected cells. Occasional cytoplasmic Daxx was never virus particle-associated. In contrast to non-infected cells, we observed a trend towards intranuclear displacement of Daxx from PML-NBs and PML clustering following infection (Figure 6A, red arrows), which could be clearly distinguished from Daxx spots in uninfected cells. This suggests that incoming viruses displace Daxx from PML-NBs by a mechanism independent of the PPxY motif of protein VI and prior to initial viral gene expression. Because we noticed occasionally large PML-NBs in infected cells, we next quantified the number of PML-NBs in wt- and M1-infected cells compared to non-infected cells. The results showed that on average, infected cells had less PML-NBs than non-infected cells, supporting our observation that PML-NBs were clustering (Figure 6B) and that the effects where PPxY motif independent. To show that the Daxx displacement from PML-NBs in the very early infection phase was caused by protein VI, we analyzed Daxx dissociation from PML-NB also in VI-wt and VI-M1 transfected cells (Figure S6). Compared to non-transfected cells, expression of protein VI-wt or VI-M1 led to translocation and cytoplasmic colocalization of Daxx (as seen in Figure 5). In addition, in several cells, Daxx was partially or completely displaced from PML-NBs and PML formed large nuclear clusters similar to those observed in infected cells (Figure S6, red arrows). We also transfected cells with expression vectors for HCMV pp71 tegument protein, known to interact with Daxx [42]. Unlike for protein VI, in pp71 transfected cells, Daxx remained nuclear and localized to some degree with PML into pp71 induced, ring-like structures also partially displacing Daxx from PML-NBs (Figure S6).

**Figure 7:**
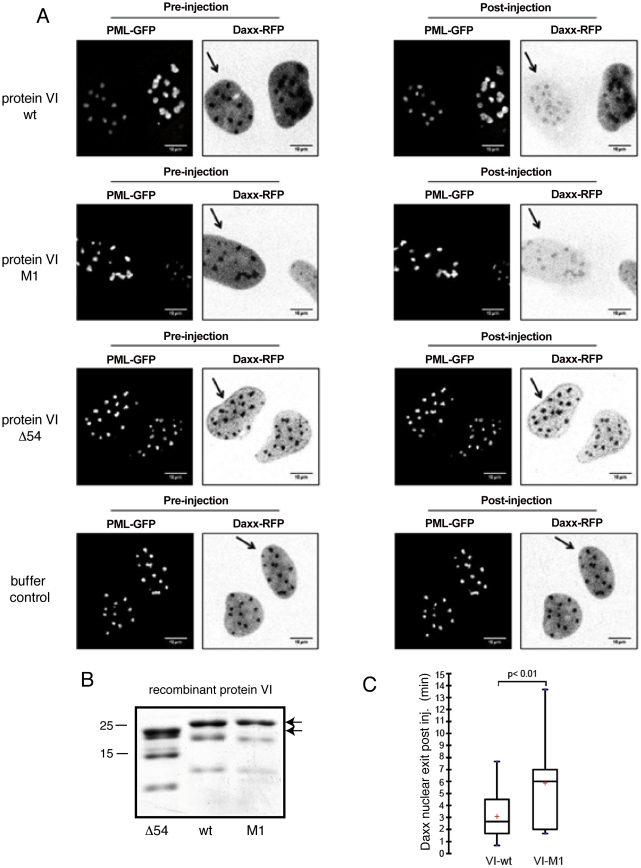
Microinjection of recombinant protein VI displaces Daxx from PML bodies and translocates it to the cytoplasm. (A) U2OS cells were cotransfected with PML-GFP (left in each panel) and Daxx-mCherry expression plasmids (right in each panel). One cell of two neighboring cells with similar expression levels was microinjected into the cytoplasm either with recombinant protein VI-wt (first row), recombinant protein VI-M1 (second row), recombinant protein delta54-VI (third row) or using a buffer control (fourth row). The left panel shows confocal images (midsection) of the cells prior to injection, the right panel shows those after approx. 6–8 min after injection. Injected cells are indicated by arrows. Note the cytoplasmic accumulation of Daxx (first and second rows). (B) The Coomassie stained gel shows the injected proteins used in A indicated by solid arrows. Movies of injections shown in (A, first to third row) are provided as supplemental Videos S1, S2, S3. (C) Quantification of nuclear export of Daxx following microinjection of recombinant protein VI-wt and VI-M1. Nuclear Daxx signal was monitored following cytoplasmic microinjection of recombinant proteins and the loss of nuclear fluorescence was plotted. The initial timepoint of Daxx export from >15 cells per condition was estimated and plotted as box-plots showing the median and average (red cross).

To directly follow Daxx displacement from PML-NBs and from the nucleus, we used microinjection of recombinant protein VI (Figure 7 and Videos S1, S2, S3). We transfected U2OS cells with Daxx-mCherry and PML-GFP expression constructs, and injected the cytoplasm with either control buffer, recombinant VI-wt or with recombinant VI-M1 (Figure 7B) and followed the distribution of Daxx-mCherry using live-cell imaging (Figure 7A). Daxx-mCherry was exclusively localized to the nucleoplasm and PML-NBs, while PML-GFP showed an intranuclear dot-like distribution with some cytoplasmic aggregates at higher levels of expression. Cytoplasmic injection of protein VI-wt or VI-M1 led to displacement of Daxx from PML-NBs and cytoplasmic accumulation of Daxx within minutes of injection (Figure 7A, first and second row compared to buffer controls in the last row). We quantified the cytoplasmic accumulation of Daxx by measuring nuclear Daxx fluorescence loss following microinjection. This quantification revealed that Daxx nuclear export occurred more rapidly post injection of protein VI-wt than VI-M1, suggesting that the PPxY motif accelerated the process of Daxx displacement (Figure 7C). Notably, Daxx displacement was paralleled by a strong increase in intranuclear mobility of PML-GFP and by fusion events between individual bodies (Videos S1 and S2), thus providing evidence that the large clustered PML-NBs, observed in fixed cells, result from the mobilization of Daxx out of the bodies.

We also microinjected recombinant protein VI (VI-delta54), lacking the amphipathic helix required for PML-NB targeting of protein VI, to see whether PML-NBs association is required for Daxx displacement. In contrast to protein VI-wt and VI-M1, injection of VI-delta54 only transiently displaced Daxx from PML-NBs and did not result in Daxx cytoplasmic translocation (Figure 7A third row and Video S3). The Daxx residence time in PML-NBs is ∼2 seconds [43]. Therefore our observation could be explained by competitive binding of VI-delta54 to Daxx, which could transiently prevent Daxx from association with PML-NBs. In summary, these data strongly suggested that protein VI from incoming adenoviral capsids can displace Daxx from PML-NBs, which in turn affects the PML-NB architecture leading to the accumulation of PML in large intranuclear clusters. Our analysis further indicate that association of protein VI with PML-NBs through the amphipathic helix is not strictly required for Daxx displacement from PML-NBs and that the PML-NB rearrangements take place prior to or are concomitant with the initiation of adenoviral transcription.

### Virion constituents from other DNA viruses can replace protein VI to promote E1A expression

**Figure 8:**
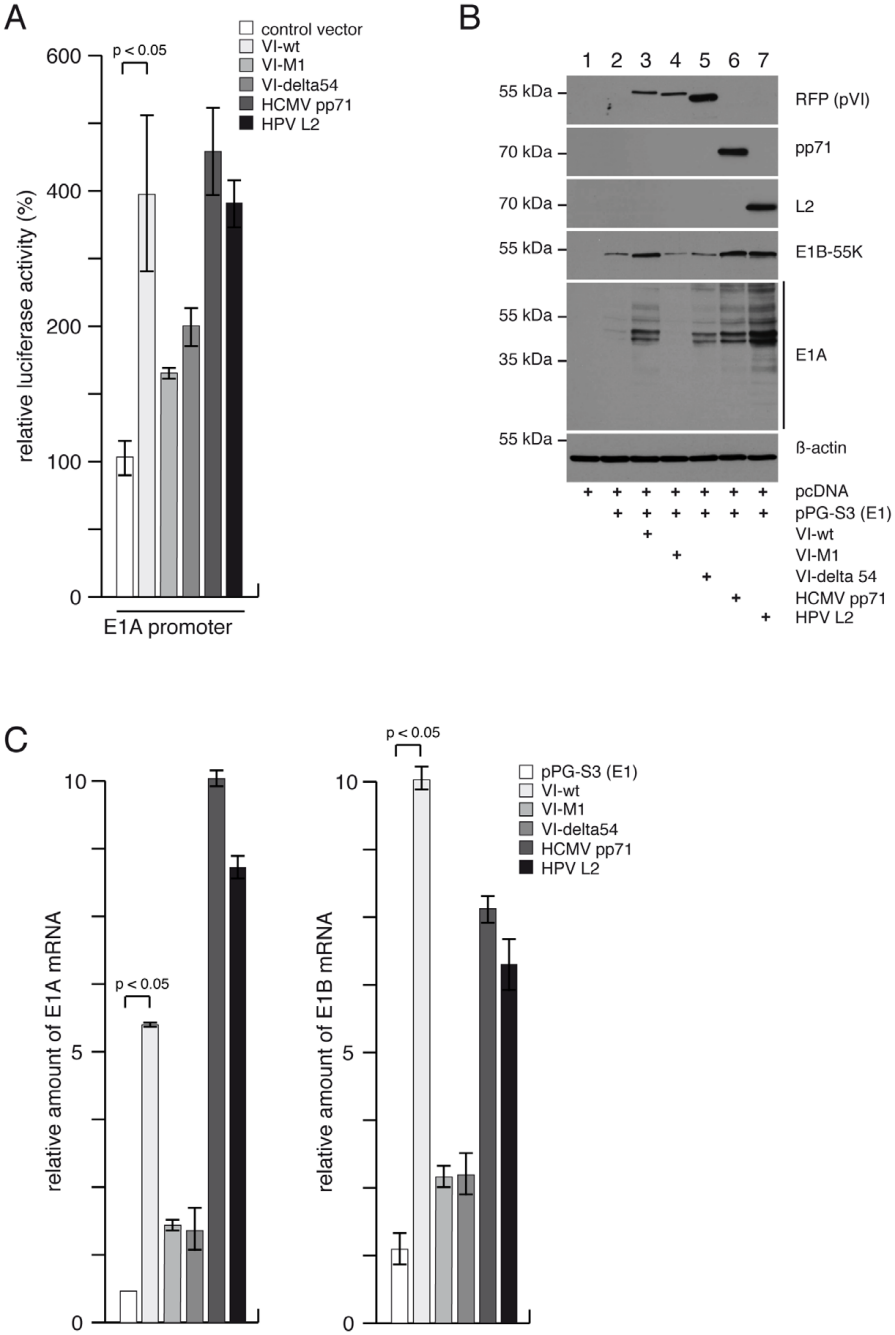
HCMV tegument protein pp71 and HPV minor capsid protein L2 stimulate E1A promoter activation. (A) H1299 cells were transfected with luciferase reporter plasmids coding for E1A promoter and effector plasmids encoding for VI-wt, VI-M1, VI-delta54, HCMV pp71, HPV L2 or an empty vector as negative control. Forty eight hours after transfection, samples were lysed and luciferase activity was measured as described before. Mean and standard deviation are from three independent experiments. (B) H1299 cells were co-transfected with plasmids containing the Ad5 E1-region (pPG-S3) and expression vector for VI-wt, VI-M1, VI-delta54, pp71 or L2. Total-cell extracts were prepared 48 h after transfection and proteins were subjected to IB using Ab against RFP (pVI), pp71 or β-actin as indicated on the right. Note that several splice variants of E1A are recognized depicted by the vertical bar. (C) Cells were transfected as in B and indicated in the legend to C. Forty eight hours after transfection total RNA was prepared from cell lysates and reverse transcribed using oligo-dT primers. E1A mRNA levels were determined using qPCR with E1A specific, exon-spanning primers. Values correspond to the mean of two experiments done in triplicates and the error bar indicates the STD.

Our data showed that protein VI activates the Ad E1 promoters by reversing Daxx repression, presumably until newly synthesized E1A can secure the Ad gene expression program. In this case, virion proteins derived from other DNA viruses known to abrogate Daxx repression should be able to substitute this function. To test this possibility, we tested whether the expression from the E1A promoter can be activated by the HCMV pp71 tegument protein or by the HPV L2 minor capsid protein, which both target Daxx [26], [44]. Similar to protein VI-wt, pp71 and L2 were able to stimulate the Ad E1A promoter (Figure 8A). Furthermore, we observed that like protein VI-wt, pp71 and L2 could also drive efficient E1A and E1B expression from a subviral construct, preserving the virus context encoding the E1A and E1B transcription units (Figure 8B, lane 3, 6 and 7). These results show that non-adenoviral virion proteins are also capable of inducing immediate early adenoviral gene expression in the absence of any further Ad protein. This induction of gene expression was through mediating transcriptional activation, as shown by elevated E1A and E1B mRNA levels (Figure 8C). Similarly, this result confirmed that elevated E1A mRNA and protein expression levels driven by protein VI require the PPxY motif, thus directly linking entry and early viral gene expression (Figure 8B, lanes 1–4). To extend the analysis for other regions of protein VI, we used the expression construct encoding protein VI-delta54, lacking the amphipathic helix, which is required to target protein VI to PML-NBs (Figure S3d). The results showed that like protein VI-M1, the construct expressing VI-delta54 only marginally stimulated the E1A promoter (compare wt-, M1 and delta54 in Figure 8A and C). In contrast, the expression of protein VI-delta54 resulted in somewhat elevated protein expression levels compared to VI-M1 suggesting that it might promote E1A expression on a post-transcriptional level. This could result from the diffuse localization of VI-delta54 in the nucleoplasm of transfected cells (compare with Figure S3). In summary, this analysis showed that efficient transcriptional activation of the E1A promoter requires the amphipathic helix in addition to the PPxY motif.

If the HCMV tegument protein pp71, that is known to remove Daxx repression from the immediate early HCMV promoter [45], activates the Ad E1A promoter, it was conceivable to speculate that protein VI would also be able to stimulate the immediate early HCMV promoter. To test this hypothesis, we constructed viral vectors encoding wt- or M1-mutated protein VI where the E1 region was replaced by a HCMV promoter controlled GFP (wt) or mCherry (M1) expression unit. We transduced U2OS cells with M1-vectors and increasing amounts of wt virus and quantified gene expression using fluorescent activated cell sorting. The results showed partial restoration of the (HCMV promoter controlled) marker gene expression from VI-M1 vector transduced cells only in cells that were co-transduced with the M1-vector and the wt-vector (Figure S7). This analysis suggested that protein VI stimulated the HCMV promoter in *trans*, like pp71 could stimulate the Ad E1A promoter in *trans* (Figure S7). Taken together the effects that protein VI has on the E1A promoter are comparable, and moreover compatible and interchangeable, with the HCMV or papillomavirus virion derived immediate early enhancing activities.

### Transactivating virion components from other DNA viruses promote Ad replication

**Figure 9:**
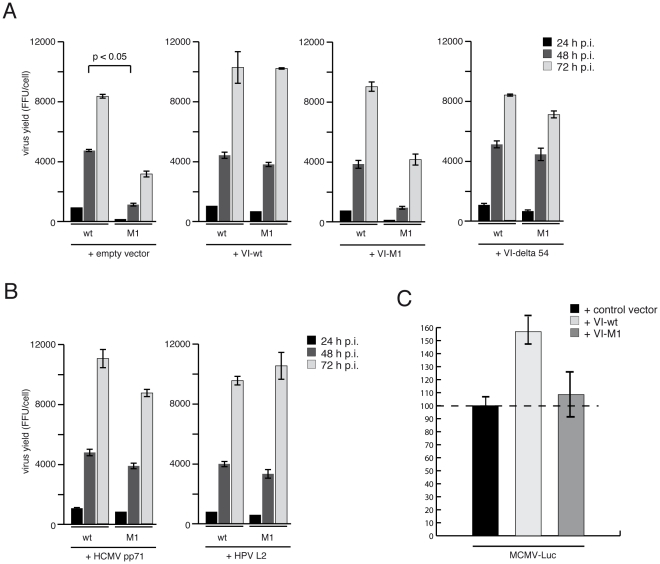
HCMV tegument protein pp71 and HPV minor capsid protein L2 can substitute transcriptional activation of the Ad genome. (A) H1299 cells were transfected with control vector, VI-wt, VI-M1 or VI-delta54 expression vector and subsequently infected with HH-Ad5-VI-wt or HH-Ad5-VI-M1 at a MOI of 50 FFU/cell. Viral particles were harvested 24, 48 and 72 h p.i. and virus yield was determined using quantitative E2A stain. (B) Experimental setup as in A including the use of the same empty vector control except that cells were transfected with expression vector for the HCMV tegument protein pp71 or the HPV small capsid protein L2. Results are from three independent experiments. (C) U2OS cells were transfected with control vectors or expression vectors for VI-wt or VI-M1 as indicated in the legend together with control expression vectors for *Renilla* luciferase. Twenty four hours after transfection, cells were infected with MCMV encoding a firefly luciferase gene controlled by the HCMV immediate early promoter. Two hours after infection cells were lysed and firefly luciferase levels were measured and normalized for renilla luciferase expression by a dual luciferase assay. Expression levels were set to 100% for empty vectors. Results are the mean of two independent experiments performed in six technical repeats. Error bars represent the STD.

Because protein VI, pp71 and L2 can stimulate Ad E1A expression independently, we next asked if they could compensate for the lack of functional PPxY motif in the replication competent HH-Ad5-VI-M1 virus. We transfected cells with expression vectors for protein VI-wt, VI-M1 and VI-delta54 (Figure 9A) and HCMV tegument protein pp71 and HPV small capsid protein L2 (Figure 9B) followed by infection with HH-Ad5-VI-wt or HH-Ad5-VI-M1 virus. The analysis showed that protein VI-wt was able to fully compensate for the M1 mutation in the virus and restored progeny virus production to wt levels, while protein VI-M1 was not able to rescue virus production and VI-delta54 resulted only in partial rescue (Figure 9A). Amazingly, HCMV pp71 and HPV L2 were also fully capable of complementing the M1 mutant virus and restored progeny virus production to wt levels (Figure 9B). Lastly, we wanted to know if the adenoviral protein VI capsid protein was also able to stimulate an immediate early promoter in the context of a non-related virus infection. We transfected U2OS cells with protein VI-wt and VI-M1 or a control vector and infected the transfected cells with a murine cytomegalovirus (MCMV) expressing luciferase under the control of the HCMV immediate early promoter (MCMV-Luc). Luciferase expression was measured 2 h after a synchronized infection to quantify the activation of the immediate early promoter. The results showed that only protein VI-wt was able to stimulate immediate early promoter in the context of MCMV infection (Figure 9C).

Taken together these results showed that protein VI promotes immediate early gene expression from the adenoviral E1A promoter, but it was also able to act on the immediate early gene expression of a non-related virus.

In summary, our analysis provides an intriguing mechanistic basis for cross genome activation of at least three unrelated DNA viruses. Our data suggest that initiation of viral gene expression can be achieved in cases where the respective virion proteins of one virus are capable of removing Daxx dependent transcriptional repression from the genome of the other virus.

## Discussion

Here, we show that the capsid protein VI is necessary for efficient initiation of Ad gene expression by activating the E1A promoter and promoting initial expression of the E1A transactivator, a function that had not been previously identified. E1A is a crucial global transcriptional activator promoting early adenoviral gene expression [37]. We show that E1A transcription and E1A protein expression at the onset of viral gene expression are reduced when cells are infected with an Ad mutant in which the PPxY motif in the capsid protein VI is inactivated. E1A mRNA production in this mutant increases with time and reaches wildtype levels, suggesting that newly expressed E1A compensates for the mutation in protein VI and drives adenoviral gene expression as soon as critical concentrations have been reached [37]. In addition, protein VI also stimulates other E1A dependent Ad promoters in the absence of any viral protein suggesting that it may act as a capsid derived E1A surrogate prior to the onset of E1A expression. Thus, protein VI is an important regulator of viral gene expression and links virus entry to the onset of gene expression. This is at least in part mediated by counteracting transcriptional repression imposed by the cellular Daxx protein and can be substituted by functionally homologous capsid proteins from unrelated DNA viruses.

In the nucleus, Daxx associates with chromatin and PML-NBs. PML-NB association with Daxx is thought to alleviate gene repression and activate apoptosis, while chromatin bound Daxx is thought to act in a transcriptionally repressive manner [7], [46], [47]. A dynamic equilibrium of Daxx between PML-NBs and chromatin association may thus govern the response status of the host cell upon infection. Moreover, an antiviral interferon response increases expression of PML and sensitizes cells for apoptosis. Artificial *knock down* of PML increases replication of Ad and other viruses, an observation that supports antiviral functions of PML [reviewed in 4], [21]. However, PML *knock down* also decreases Daxx steady state levels by an unknown mechanism, showing that antiviral activity might be mediated by Daxx rather than PML [21]. This would be in line with our observation that Daxx *knock down* has much stronger pro-replicative effects on Ads.

Here we demonstrate that Daxx directly represses Ad E1 promoters. So far, it has been shown that Daxx inactivates the major immediate early promoter of HCMV [45], is recruited to HSV genomes via SUMO dependent pathways [48] and is likely to associate with incoming avian sarcoma virus (ASV) and human immunodeficiency virus (HIV) genomes [49], [50]. Therefore, Daxx could act as a cytoplasmic and/or nuclear DNA sensor and may be part of a cellular innate defence mechanism against DNA virus infection (or other pathogens) by simply assembling repressive complexes on incoming DNA [51]. This is supported by two recent studies showing that Daxx selectively represses procaryotic DNA expression [52] and that frequent epigenetic silencing of integrated retroviral genomes could be reversed by Daxx depletion, showing epigenetic control of pathogen DNA by Daxx associated mechanisms [53]. Daxx mutants that fail to associate with the HSV genome also fail to induce repression on the HSV genome, underlining the important role of Daxx as part of the cellular innate antiviral defence mechanism [48].

If Daxx serves in antiviral intrinsic immunity to repress viral genomes, virion proteins are viral countermeasures. Several structural proteins from viral particles have been reported to interact with Daxx, including tegument protein pp71 [HCMV]; [ 42,54], minor capsid protein L2 [HPV; 26], DENVC [Dengue virus; 55], p6 [HIV GAG; 56], nucleocapsid protein PUUV-N [Hantavirus; 57], Integrase [ASV], [ HIV; 49,53] and protein VI (Ad, this study).

The best studied is the tegument protein pp71 of HCMV, which enhances infectivity and replication through activation of the immediate early promoter. This requires colocalization of the viral genome with PML-NBs and Daxx degradation via pp71 [23], [42], [44], [58], [59]. In addition, pp71 was also shown to activate gene expression from HSV-1, a different herpesvirus, showing that its function is not restricted to HCMV [60]. Unlike for HCMV, degradation of Daxx [through E1B-55K; 21] during Ad infection requires early gene expression. Here we observe quantitative removal of Daxx from PML-NBs upon infection without degradation before gene expression is established. We propose that this is caused by protein VI derived from the entering capsid, which partially associates with PML-NBs during entry. Similar to what we observe early in infection, transfected protein VI also displaces Daxx from PML-NBs and translocates it into the cytoplasm. Similarly, microinjected protein VI leads to rapid exclusion of Daxx from PML-NBs and cytoplasmic accumulation suggesting active removal following protein VI nuclear import. Deletion of the N-terminal amphipathic helix from protein VI, which serves as PML-NB targeting domain, still mediated the transient dissociation of Daxx from PML-NBs suggesting that competitive binding and a short residence time of Daxx in PML-NBs can also cause Daxx removal from PML-NBs [43]. Daxx depletion from PML-NBs also provokes intranuclear mobility and clustering of PML, reminiscent of infected cells and showing that Daxx contributes to the integrity of PML-NBs, which confirms previous observations [10].

Ad-wt, but not a virus with the mutated PPxY-motif in protein VI, counteracts Daxx repression for efficient viral gene expression. Protein VI wt also induces a more rapid Daxx displacement from PML-NBs and subsequent nuclear export than its mutated counterpart. In contrast, binding of Nedd4-family ubiquitin ligases to the PPxY of protein VI abolished cytoplasmic translocation of Daxx at steady state, suggesting that Nedd4 binding to protein VI competes with the interaction between Daxx and protein VI. Increasing the efficiency of Daxx mobilization in the nucleus, and simultaneously preventing Daxx nuclear export or limiting the time Daxx resides in the cytoplasm through competitive binding to Nedd4, could lead to efficient derepression and prevent Daxx from activating apoptosis (via JNK pathways), which could explain why Nedd4 binding is beneficial for the virus [10], [61].

Displacing Daxx from PML-NBs immediately after virus entry prevents antiviral apoptotic processes, possibly increasing Daxx mediated repression by epigenetic silencing [reviewed in 5]. We observe Daxx removal from PML-NBs for wt- as well as M1 mutated protein VI. In contrast, only wt-VI shows a strong stimulation and direct association with viral E1 promoters as determined by ChIP. In addition, proper transcriptional activation of the E1A promoter required the presence of the amphipathic helix. Thus, reversal of Daxx repression by protein VI from viral promoters might provide an additional explanation for Nedd4 function and the role of the PPxY motif. Targeting Nedd4 to viral promoters via the PPxY could result in ubiquitylation of histone or the histone-like DNA bound viral protein VII or other Daxx interactors, to open the chromatin structure for transcription.

In this scenario, protein VI would prevent formation or disassemble already bound repressive complexes from viral promoters via the PPxY motif and Nedd4. This would explain why the M1 mutant still displaces Daxx from PML-NBs, but retains only a minor capacity of stimulating viral gene expression presumably through interfering with the assembly of new Daxx repressive complexes. This model would also support the observation that, like protein VI-M1, protein VI without amphipathic helix (but intact PPxY and still capable of Daxx binding) hardly stimulates the E1A promoter. This mutant is diffusely distributed in the nucleus showing that the helix contributes to proper intranuclear targeting of protein VI. Mislocalization therefore could reduce the capacity to remove or prevent assembly of Daxx repressive complexes on the E1A promoter. How this mutant still retains some capacity of stimulating E1A protein expression (and as a consequence partially rescues the M1-virus) without activating the E1A promoter is currently unclear.

**Figure 10:**
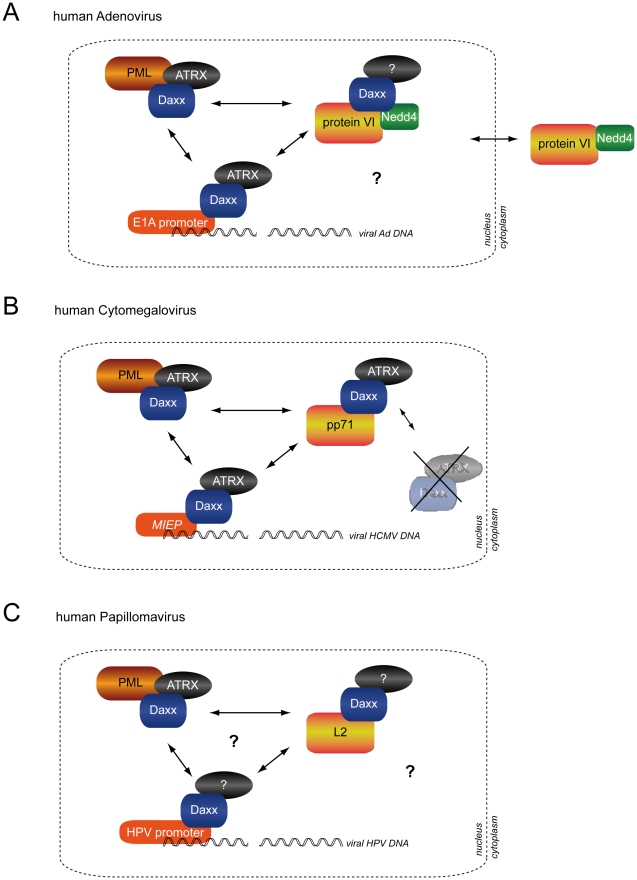
Model for genome activation of DNA viruses through structural proteins of the virion. A schematic representation of Daxx restriction mediated by human adenovirus capsid protein VI (A), HCMV tegument protein pp71 (B) and HPV minor capsid protein L2 (C). Daxx repressive complexes (containing the two transcriptional repressor Daxx and ATRX) assemble at PML-NBs and/or on viral genomes. Transcriptional activation of the viral genome requires removal of Daxx repressive complexes and/or the prevention of their assembly possibly involving PML-NBs. (A) The adenoviral E1A promoter is activated through targeting of Daxx by the adenoviral capsid protein VI and presumably Nedd4 ligases. This requires an N-terminal amphipathic helix and a conserved PPxY motif on protein VI. The latter is required for binding and nuclear targeting of Nedd4 ligases. As a consequence Daxx is not degraded but likely is displaced from the viral genome and PML-NBs thereby removing and preventing assembly of new Daxx repressive complexes on the viral genome. The fate of the removed complexes including ATRX is currently not clear. (B) For HCMV the major immediate early promoter (MIEP) is activated through tegument protein pp71, which binds to Daxx and mediates its proteasomal degradation thus removing ATRX and preventing Daxx repressive complexes on the viral genome. (C) The major HPV promoter is repressed by Daxx related mechanisms although very little is currently known. Daxx is targeted by the minor capsid protein L2, which also modulates PML-NBs. However, a formal role in Daxx de-repression of the viral promoter or a role for ATRX has not been established. Nevertheless, we show in this report the cross-activation of the adenoviral E1A promoter by pp71 and L2 and the activation of the MIEP by protein VI suggesting a common principle of genome activation.

Removal of Daxx by components of incoming virions to initiate gene expression is a common viral strategy. Our experiments are the first to show that the consequences are not virus-family specific, but provoke global changes in transcriptional activity that allow transcriptional activation of one viral genome (here the Ad or MCMV genome) by the virion protein of unrelated viruses (here pp71 from HCMV and L2 from HPV; or the CMV promoter by protein VI). All three virion proteins (VI, pp71 and L2) target Daxx repressive complexes. The details of these interactions are not fully understood but they share similarities as highlighted in the model in Figure 10. We suggest that activation of viral gene expression for the three viral systems (Ad, HCMV and HPV) involves prevention and removal of Daxx repressive complexes. This is achieved by preventing Daxx-PML interaction or association of Daxx repressive complexes with the viral genome and (in some cases) involves the degradation of components of the complex (Figure 10). Neither pp71 nor L2 contain a PPxY motif suggesting different modes of action on Daxx or components of the Daxx repressive complex. Protein VI is also not restricted to Ads in its de-repressive activity and is able to stimulate the immediate early HCMV promoter. Several other viral capsid proteins have been reported to encode PPxY motifs [reviewed in 62]. The research focus for those motifs has been on their role in virus budding despite the presence of these proteins in the capsids of many viruses during virus entry. If virion derived PPxY (and related motifs) are part of a more general activation mechanism for several viruses then this could also mean that co-infections with different viruses, frequently observed *in vivo*, could promote each other. Similarly it is an interesting question, whether superinfections of a latently infected cell by another de-repressive virus would support reactivation of the latent genome. Epidemiological data from a recent study show that Ad/HCMV co-infections *in vivo* happen as often as mono-infections and the authors suggest that this could reflect co-viral reactivation [63]. Our data would provide a mechanistic basis for this observation, which is potentially applicable to several types of viral co-infections.

Lastly, we believe that gene regulatory functions of viral structural proteins should be considered when addressing safety issues for the application of viral vectors (e.g. adenoviral vectors) in therapeutic settings where (re)activation of unrelated (latent) viruses is unwanted.

## Materials and Methods

### Cell culture

U2OS, H1299 and HepaRG cells were grown in Dulbecco's modified Eagle's medium supplemented with 10% fetal calf serum (FCS), 100 U of penicillin, 100 µg of streptomycin per ml in a 5% CO_2_ atmosphere at 37°C. For HepaRG and HAD (Daxx *knock down*) cells media was supplemented with 5 µg/ml of bovine insulin and 0.5 µM of hydrocortisone [20], [21].

### Transfections and luciferase reporter assays

Tagged protein VI, PML, Daxx and Nedd4 expression vectors have been described previously [36], [64]. E1A was expressed from constructs encompassing the left part of the viral genome including left inverted terminal repeat (ITR) and the E1 genes (pPG-S3). N-terminal flag-tagged human PML-isoforms I-VI were expressed from pLKO.1-puro vector (kindly provided by R. Everett). Codon optimized HPV (type 16) L2 expression vector was kindly provided by M. Mueller, DKFZ Heidelberg. Expression vector pCGN71 [65] encodes an *Xba*I-*Bam*HI PCR fragment 0corresponding to the HCMV strain AD169 UL82. Dual luciferase assays were performed according to manufacturers instructions and have been described previously [66]. Promoter constructs are based on the pGL3-basic vector (Invitrogen, cloning details will be provided upon request).

### Viruses

E1-deficient viral vectors BxAd5-VI-wt-GFP and BxAd5-VI-M1-mCherry are based on human Ad serotype 5 and have been cloned using homologous and site-specific recombination using bacterial artificial chromosomes (BACs) as described in detail recently [36]. Replication competent wt virus HH-Ad5-VI-wt is identical to the previously described H5*pg*4100 [67]. The virus mutant HH-Ad5-VI-M1 carries an altered PPxY motif in the protein VI open reading frame [PPSY = >PGAA; Fig. S1; [36]]. Viruses were constructed, propagated and titrated on HEK293 cells as detailed in Figure S1.

### Indirect immunofluorescence and protein analysis

For immunofluorescence analysis cells were washed in PBS and fixed for 20 min using 4% paraformaldehyde. Detection of endogenous antigens using primary and secondary Ab was done in IF-buffer (PBS with 10% FCS and 0.2% Saponin) followed by washing and embedding in Prolong Gold (Invitrogen). A list of primary and secondary Ab used in this study is given in Protocol S1 in Text S1. Images are presented as maximum image projections if not indicated otherwise. For protein analysis total-cell lysates were prepared and analyzed by western blot using standard protocols. The list of the antibodies used in this study and details for immunoprecipitation (IP) procedures are given in Protocol S1 and Protocol S2 in Text S1.

### ChIP assay and quantitative real-time (qRT) PCR analysis

H1299 cells were infected with HH-Ad5-VI-wt or HH-Ad5-VI-M1 at 50 fluorescence forming units/cell (FFU/cell) and harvested 24 h p.i. ChIP analysis was performed as described previously with some modifications [68], [69]. For ChIP, proteins from 2×10^6^ cells were cross-linked to DNA with 1% formaldehyde in PBS for 10 min at room temperature. The reaction was quenched and cells were washed with PBS and harvested by scraping off the dish. Nuclei were isolated by incubation of cross-linked cells with 500 µl buffer I (50 mM Hepes-KOH, 140 mM NaCl, 1 mM EDTA, 10% glycerol, 0.5% NP-40, 0.25% Triton X-100) for 10 min on ice and pelleted by centrifugation. The nuclei were subsequently washed with 500 µl buffer II (10 mM Tris-HCl, 200 mM NaCl, 1 mM EDTA, 0.5 mM EGTA), pelleted again and resuspended in 500 µl buffer III (1% SDS, 10 mM EDTA, 50 mM Tris-HCl). Chromatin was fragmented by sonication using a Bioruptor (Diagenode) to an average length of 100–300 bp. After addition of 10% Triton X-100, cell debris were pelleted by centrifugation (20,000× g, 4°C) and supernatants were collected. Chromatin was diluted with dilution buffer (0.01% SDS, 1.1% Triton X-100, 1.2 mM EDTA, 16.7 mM Tris-HCl, 167 mM NaCl). To reduce non-specific background, chromatin was pre-incubated with salmon-sperm DNA protein-A agarose beads (Upstate). Antibodies were added and incubated for 16 h at 4°C. Fifty µl agarose beads were added to precipitate the chromatin-immunocomplexes for 4 h at 4°C. Beads were washed once with low-salt buffer (0.1% SDS, 1% Triton X-100, 2 mM EDTA, 20 mM Tris-HCl, 150 mM NaCl), once with high-salt buffer (0.1% SDS, 1% Triton X-100, 2 mM EDTA, 20 mM Tris-HCl, 500 mM NaCl), once with LiCl-wash buffer (0.25 M LiCl, 1% Nonidet P-40, 1% Na-deoxycholate, 1 mM EDTA, 10 mM Tris-HCl) and twice with TE buffer. Chromatin was eluted from the beads in elution-buffer (50 mM Tris-HCl pH 8.0, 10 mM EDTA, 1% SDS) for 10 min at 95°C. Proteinase K was added for protein degradation and samples were incubated for 1 h at 55°C. For preparation of input controls, samples were treated identical to IP samples except that non-specific Ab were used. qPCR analysis was performed using a Rotor Gene 6000 (Corbett Life Sciences, Australia) in 0.5 ml reaction tubes containing 1/100 dilution of the precipitated chromatin, 10 pmol/µl of each synthetic oligonucleotide primer (E1A fwd 5′TCCGCGTTCCGGGTCAAAGT3′; E1A rev5′GTCGGAGCGGCTCGGAG3′; E1B fwd 5′GGTGAGATAATGTTTAACTTGC3′ E1B rev 5′TAACCAAGATTAGCC CACGG3′), 5 µl/sample *SYBR Green PCR Master Mix* (Applied Biosystems). The PCR conditions used: 7 min at 95°C, 45 cycles of 12 s at 95°C, 40 s at 60°C and 15 s at 72°C. The average Ct-value was determined from triplicate reactions and normalized against non-specififc IgG controls with standard curves for each primer pair. The identities of the products obtained were confirmed by melting curve analysis. For qPCR analysis, U2OS cells were infected with 1, 10 and 200 physical particles/cell and genome copy numbers were determined in nuclear and cytoplasmic fractions using hexon specific primers [70].

### Extraction and quantification of newly transcribed RNA

4sU (Sigma) was added to the cell culture media for 1 h, made up to a final concentration of 200 µM, during indicated time points throughout infection. Cells were harvested using Trizol reagent (Invitrogen) and total RNA isolated by phenol-chloroform extraction. Biotinylation and purification of 4sU-tagged RNA (newly transcribed RNA), was performed as described previously [71]. Five hundred ng of each newly transcribed RNA per reaction was reverse transcribed in 25 µl reactions using Superscript III (Invitrogen) and oligo-dT primers (Invitrogen) following the manufacturer's instructions. PCR was performed on a Light Cycler (Roche Molecular Biochemicals). Each reaction, every sample in duplicates, was carried out using 5 µl of cDNA (1∶10 dilution) and 15 µl reaction mixtures of Quantitect SYBR Green PCR master mix and 0.5 µM of the primers. PCRs were subjected to 10 min of 95°C hot-start, and SYBR Green incorporation was monitored for 45 cycles of 95°C denaturation for 10 s, 58°C annealing for 3 s, and 72°C elongation for 10 s. The data were analyzed using the ΔΔCt method using GAPDH as an endogenous reference, and the mock-infected sample as a calibrator. Values were normalized to 100% for wt-infected cells. The E1A 13S mRNA specific and the GAPDH specific primers were described in [72]. Primers used are listed below: E1A13S-fwd (5′-GGC TCA GGT TCA GAC ACA GGA CTG TAG), E1A13S-rev (5′-TCC GGA GCC GCC TCA CCT TTC), GAPDH-fwd (5′-TGG TAT CGT GGA AGG ACT CA), GAPDH-rev (5′-CCA GTA GAG GCA GGG ATG AT).

### Microinjection and protein purification

Details for microinjection are given in the Figure 8 and video legends (Video S1). Briefly, U2OS cells were cotransfected with PML-GFP and Daxx-mCherry expression plasmids and cultivated on a heated stage (37°C) in CO_2_ stabilized medium attached to a SP5 confocal microscope (Leica) equipped with a microinjection device (Eppendorf). Microinjected cells were imaged within a single confocal plane at the nuclear midsection at 20 s intervals for 10 frames prior to injection and 40 frames post injection. Injected proteins were purified as His-tagged proteins using standard procedures and dialyzed into transport buffer as detailed previously [30], [36].

### Statistical analysis

Data are presented as mean, error bars as standard deviation (STD). Statistical analysis was done using paired students t-test except for Figure 6B where a two-tailed two sample t-test was used. The p-values are indicated.

### List of accession numbers for proteins used in this study

Human Daxx **CAG33366.1**, Protein VI **AAA96411.1**, Human Adenovirus Type 5 **HY339865**, PML-I **AAG50180**, PML-II **AF230410**, PML-III **S50913**, PML-IV **AAG50185**, PML-V **AAG50181**, PML-VI **AAG50184**, HCMV pp71 **ACZ79993.1**, humanized HPV L2 (HPV16).

## Supporting Information

Figure S1 **Construction of virus mutant HH-Ad5-VI-M1 by site directed mutagenesis.** (A) For the construction of the replication competent virus mutant HH-Ad5-VI-M1, the Ad5 wild type genome in HH-Ad5-VI-wt [H5pg4100; 67] was inserted into the *Pac*I site of the bacterial cloning vector pPG-S2 [67]. It lacks nucleotides (nt) 28593 to 30471 (encompassing most of E3) and contains an additional unique endonuclease restriction site at nt 30955 (*Bst*BI) (nucleotide numbering is according to the published Ad5 sequence from GenBank, accession no. AY339865). In vitro mutagenesis was used to introduce the M1 mutation into the transfer vector pL3 containing the protein VI gene. The resulting transfer vector pL3-M1 was used to replace the *Sgf*I-*Pme*I fragment in the genome encoding plasmid to generate HH-Ad5-VI-M1. For generation of the HH-Ad5-VI-M1 and the wt control virus, infectious viral DNA was released from the recombinant plasmids by *Pac*I digestion and transfected into the complementing cell line 2E2 [73]. Viral progeny was amplified in 2E2 cells followed by purification on CsCl_2_ gradients. The integrity of the recombinant virus was verified by restriction digest and DNA sequencing of the entire protein VI gene from isolated viral DNA. (B) For subsequent infection experiments, virus stocks were titered on HEK293 cells [74] and fluorescent forming units were determined by E2A stain for viral replication centers. Virus growth was determined by harvest of infected cells at 24, 48 and 72 h p.i. followed by three freeze/thaw cycles. The cell lysates were serially diluted and virus yield was determined by quantitative E2A stain, 24 h after infection of HEK293 cells as described previously [75]. Viral supernatants were normalized for infectious units (e.g. 50 fluorescence forming units, FFU) prior to use in experiments showing roughly four fold higher ratio of infectious to non infectious particles for the HH-Ad5-VI-wt compared to HH-Ad5-VI-M1.
(TIF)

Figure S2 **Quantification of viral genomes in fractionated cells.** (A) U2OS cells were synchronously infected with replication competent HH-Ad5-wt or HH-Ad5-M1 virus at 200, 10 or 1 physical particles per cell (pp/cell) as virus input –A-. Forty five min after infection, the cytoplasmic –B- and nuclear –C- fractions were separated using nucleo-cytoplasmic fractionation according to the manufacturer's protocol (Pierce). Each fraction was subjected to extraction of the adenoviral genomes using the high pure viral nucleic extraction kit (Roche) according to the manufacturer's instructions. The viral genomes were quantified in –A-, -B- and –C- by qPCR using AQ1 and AQ2 oligonlucleotides to amplify part of the hexon gene [described in 70]. Serial dilutions of a pcDNA3.1 plasmid coding for the Ad5 hexon were used to obtain the standard curve for quantification. The copy number of viral genomes of each fraction was calculated from the C_t_-values obtained for each sample. Values were used to determine the cell-associated viral genomes and expressed as percentage of virus input (1 = 100×(B+C/A) reflecting cell binding and virus entry capacity. The percentage of nuclear-associated genomes was calculated and expressed as percentage of total cell-associated genomes (2 = 100×(C/B+C) This value represents viral genomes associated with the nuclear fraction after transport towards the nucleus. To identify how many genomes initiate replication, cells were infected in parallel and stained for replication centers at 24 h p.i. using E2A specific Ab. E2A positive cells were counted and the percentage of E2A positive cells was calculated –D-. In order to discriminate between a viral expression defect and a decrease in virus nuclear transport and accumulation that could account for a loss of expression, the percentage of E2A positive cells was normalized with the percentage of nuclear-associated genome (3 = D/2). To quantify the M1 defects, both the cell-and nuclear-associated genomes of the mutant and the E2A expression were compared to the wt values and presented as percentage of wt. (B) Quantification of intracellular viral genomes and genome replication as in A. Cell-associated genomes are the sum of nuclear and cytoplasmic fractions normalized for total viral input while nuclear-associated genomes were normalized to cell-associated genomes. Twenty four hours p.i., the expression of the E2A marking viral replication was quantified in parallel infected cells and the percentage of E2A positive cells was normalized for nuclear-associated genomes. All data for M1 are expressed as percentage of the HH-Ad5-pVI-wt virus ( = 100%). * mean value (+/− STD) from independent experiments at 200, 10 and 1 pp/cell (each done in triplicates). Note that differences are MOI independent except for E2A expression. Reduction of M1 virus in the cell associated fractions was likely due to post-endosomolytic degradation because previous work showed equal efficiency for both viruses in membrane lysis [36]. The additional reduction of the M1 virus in the nucleus-associated fraction was independent of the pp/cell ratio and reflects nuclear accumulation defects, as observed for the M1 non-replicative virus in our previous study [36]. The reduced initiation of replication for the M1 virus at low, but not high MOIs, explains why production yields and virus amplification of the M1 mutant virus is unaffected at high pp/cell ratios while up to 20-fold reduced infection rates can be observed at low pp/cell ratios [Fig. 1; [36].
(TIF)

Figure S3 **PML-NB association of protein VI requires the amphipathic helix.** To identify the domain of protein VI required for PML-NB association, several mRFP tagged constructs for protein VI were transfected into U2OS cells and stained for association with endogenous PML. The mRFP signal is shown in the left column. An overlay of the mRFP protein VI signal (red), endogenous PML (green) and the nuclear envelope stained with MAb 414 (Abcam) against the nuclear pore complex (grey) is shown in the right column. Association of protein VI and PML is depicted by a white arrow and magnified in the top right corner as inset to each overlay panel. Transfected constructs with the functional domains amphipathic helix, nuclear localization signal (NLS) and PPxY motif and their respective modification are indicated to the left. Top to bottom : full length wt protein VI as used in the transfections in Figure 2B (a), C-terminal processed wt protein VI (b), as b with mutated PPxY motif (c), processed protein VI with deleted amphipathic helix (delta 54, d), processed protein VI with two essential tryptophan residues mutated [W37/41]; [ 31] in the amphipathic helix (e) and the same construct as full length version (f). This analysis confirmed that protein VI is targeted to PML-NBs and localized in close proximity to PML. Association of protein VI with PML-NBs was not affected when the PPxY motif was mutated (c) or when processed protein VI, as it is present in the entering virus, was used (b). However, mutating or deleting the amphipathic helix of protein VI changes its distribution from a dot-like pattern towards a partial or complete diffuse, predominant nuclear localization and with loss of its PML-NBs association (d, e, f). These data indicate that the amphipathic helix was a major determinant in targeting protein VI towards PML-NBs. Note that clustering of endogenous PML-NBs in the transfected cells still occurs when the amphipathic helix is mutated or deleted.
(TIF)

Figure S4 **Protein VI mediates adenovirus transcriptional activation of all Ad promoters.** Subconfluent H1299 cells were transfected with luciferase reporter plasmids encoding for the E1A-, E1B-, pIX-, E2early-, E2late-, E3-, E4-promoters and the major late promoter (MLP) and effector plasmids expressing VI-wt, VI-M1. Fortyeight hours after transfection, samples were lysed and absolute luciferase activity was measured as described by the manufacturer (*dual luciferase kit/Promega*). The luciferase activity of each individual promoter was normalized to 100%. The means are presented for three independent experiments. Error bars represent STD. The results show that protein VI stimulates all adenoviral promoters between ∼1.5 to ∼4 fold independent of any other adenoviral protein. Most stimulation is achieved by protein VI-wt compared to protein VI-M1. This is an indication that Daxx repressive mechanisms largely control adenoviral gene expression and that protein VI is an important transactivator that requires the PPxY motif to be fully active.
(TIF)

Figure S5 **Protein VI targets Nedd4 ligases to PML-NBs via the PPxY motif.** U2OS cells were transfected with expression constructs for GFP-tagged Nedd4 ligases and RFP-tagged expression constructs for protein VI-wt or VI-M1 and stained for endogenous PML, as indicted to the left of each row. From top to bottom; Nedd4.1-GFP was cotransfected with VI-wt (a) or VI-M1 (b), Nedd4.2 was cotransfected with VI-wt (c) or VI-M1 (d) or VI-wt was cotransfected with a catalytical inactive mutant of Nedd4.2 (TD, e). An overlay of endogenous PML (grey, first column), Nedd4 (green, second column) and VI (red, third column) is shown in the fourth column. The small inset in each panel shows a magnification of the grey box in the overlay, highlighting colocalization of the three proteins at PML-NBs. Please note that only VI-wt, but not VI-M1, targets Nedd4 ubiquitin ligases to PML-NBs irrespective of the ligase activity. This analysis shows that Nedd4 ligases can be efficiently imported into the nucleus and targeted to PML-NBs, by binding to the PPxY motif of protein VI. We were unable to show that during Ad entry Nedd4 is also translocated into the nucleus or towards PML-NBs due to the bad quality of existing Nedd4 Ab. The observation that transfected protein VI can translocate transfected Nedd4 into the nucleus raises the possibility that incoming particles could also relocate Nedd4 ligases towards the nucleus through association with capsid associated protein VI and alter physiological functions and/or exploit Nedd4 family members to initiate and promote viral replication.
(TIF)

Figure S6 **Transfected viral capsid proteins partially displace Daxx from PML bodies.** H1299 (a–c) and U2OS (d–f) cells were transfected with either empty control plasmid (a, d) or mRFP-tagged VI-wt (b, e) or mRFP-tagged VI-M1 (c) or with an HA-tagged expression vector for the pp71 tegument protein of the human cytomegalovirus (f, all first columns). Transfected cells were stained for endogenous Daxx (second column) and endogenous PML (third column). The localization of capsid proteins was determined using the RFP signal for VI-wt and VI-M1 or using Ab against the HA-tag to detect pp71 tegument protein. An overlay of all three signals is shown in the last column were capsid proteins are shown in white, Daxx in green and PML in red. Note that red arrows point at PML without (b, c) or with reduced (d) Daxx colocalization or at pp71 induced nuclear structures recruiting Daxx and PML (f). This analysis shows that protein VI (wt and M1) alone is capable of displacing Daxx from PML-NBs and support that protein VI is responsible for the observations made in Figure 6, which show that adenovirus infection results in displacement of Daxx from PML-NBs prior to gene expression. Using different cell lines further supports that protein VI mediated displacement of Daxx from PML-NBs is a genuin property of protein VI. We observed that PML-NB displacement and cytoplasmic accumulation of Daxx was most efficient in H1299 cells while in U2OS cells Daxx was less prominently associated with PML-NBs at steady-state and also less prominently translocated to the cytoplasm upon VI-wt expression. In contrast, expression of VI-M1 let to very efficient Daxx translocation and cytoplasmic colocalization in all three cell lines tested. The observed clustering of PML following transfection is reminiscent of the induced mobility and fusion observed for transfected PML after Daxx displacement in cells microinjected with protein VI as it is shown in Figure 7 and Videos S1 and S2.
(TIF)

Figure S7 **Protein VI-wt activates the CMV promoter of E1-deleted Ad vector particles with M1 mutated protein VI.** U2OS cells were transduced with 1 physical particle per cell (pp/cell) of E1-deleted viral vector BxAd5-VI-M1-mCherry (expressing mCherry under CMV promoter control and M1 mutated protein VI) and different amounts of viral vector BxAd5-VI-wt-GFP (expressing GFP under CMV promoter control and wt protein VI). The ratios of M1- to wt-virus are indicated on the x-axes (values in pp/cell). Transduction levels were determined by FACS and are shown separately for M1 (mCherry, light grey bars) and wt (GFP, dark grey bars). The dotted lines indicate wt transduction levels at 1 pp/cell or M1 transduction levels at 1 pp/cell as indicated to the right of the graphic. The increased transduction levels with the M1-virus co-incided with co-transduction of wt-vectors (data not shown). The data show that expression of (CMV-promoter-driven) mCherry from the genome of the E1-deleted Ad vector that encodes protein VI with the M1 mutation is restored when the same cell is also transduced with wt-vector particles that contain protein VI-wt. This observation supports a role for protein VI in activating the CMV promoter and shows that an adenoviral protein (capsid protein VI) can activate the early promoter of a non-related DNA virus (immediate early promoter of HCMV). Because Daxx represses the CMV promoter [45], transactivation by protein VI presumably occurs through removal of Daxx repression.
(TIF)

Text S1 **The supporting information contains a list of all antibodies used in this study (Protocol S1), a detailed protocol for the co-immunoprecipitation assays (Protocol S2) and additional references used in Figures S1, S2, S3, S4, S5, S6, S7.**
(DOC)

Video S1 **Microinjection of recombinant protein VI-wt displaces Daxx from PML bodies to the cytoplasm.** U2OS cells were cotransfected with Daxx-mCherry (left panel) and PML-GFP (middle panel) expression plasmids (superimposed signal on the right) and cultivated at 37°C on a heated stage in CO_2_ stabilized medium attached to a SP5 confocal microscope equipped with a microinjection device (Eppendorf). The left cell of two cotransfected cells with equal expression levels of both proteins was then microinjected into the cytoplasm using recombinant protein VI at a final concentration of 0.3 µg/µl. Prior to injection, cells were imaged using the SP5 confocal microscope at 20 s intervals for 10 frames at a single optical section with pinhole setting of two using a 20× magnification and maximum resolution. Injection was performed manually under optical control in DIC mode and immediately after images were taken at 20 s intervals for further 40 frames without changing the optical setting to minimize the time between injection and imaging (time delay ∼1–2 min). Note the cytoplasmic accumulation of the Daxx signal following injection.
(AVI)

Video S2 **Microinjection of recombinant protein VI-M1 displaces Daxx from PML bodies to the cytoplasm.** U2OS cells were cotransfected with Daxx-mCherry (left panel) and PML-GFP (middle panel) expression plasmids (superimposed signal on the right) and cultivated at 37°C on a heated stage in CO_2_ stabilized medium attached to a SP5 confocal microscope equipped with a microinjection device (Eppendorf). The left cell of two cotransfected cells with similar expression levels of both proteins was then microinjected into the cytoplasm using recombinant bacterially expressed and purified protein VI-M1 at a final concentration of 0.3 µg/µl. Prior to injection, cells were imaged using the SP5 confocal microscope at 20 s intervals for 10 frames at a single optical section with pinhole setting of two using a 20× magnification and maximum resolution. Injection was performed manually under optical control in DIC mode and immediately after images were taken at 20 s intervals for further 40 frames without changing the optical setting to minimize the time between injection and imaging (time delay ∼1–2 min). Please note the increase of PML intranuclear dynamics in the injected cell that starts as soon as the Daxx signal diminishes. This dynamic was seen in all cells injected with VI-wt or with VI-M1 but never in buffer injected control cells.
(AVI)

Video S3 **Microinjection of recombinant protein VI-delta54 transiently displaces Daxx from PML bodies but fails to export Daxx to the cytoplasm.** U2OS cells were cotransfected with Daxx-mCherry (left panel) and PML-GFP (middle panel) expression plasmids (superimposed signal on the right) and cultivated at 37°C on a heated stage in CO_2_ stabilized medium attached to a SP5 confocal microscope equipped with a microinjection device (Eppendorf). The left cell of two cotransfected cells with similar expression levels of both proteins was then microinjected into the cytoplasm using recombinant bacterially expressed and purified protein VI-delta54 at a final concentration of 0.3 µg/µl. Prior to injection, cells were imaged using the SP5 confocal microscope at 20 s intervals for 10 frames at a single optical section with pinhole setting of two using a 20× magnification and maximum resolution. Injection was performed manually under optical control in DIC mode and immediately after images were taken at 20 s intervals for further 40 frames without changing the optical setting to minimize the time between injection and imaging (time delay ∼1–2 min). Please note that Daxx temporarily is displaced from PML-NBs (∼0–3 min post-injection) followed by a return to PML-NBs. During Daxx displacement please also note the increase of PML intranuclear dynamics in the injected cell that diminishes with the return of Daxx to the PML-NBs.
(AVI)
