# Supplementary figures and images for: Transcriptional Activation of the Adenoviral Genome Is Mediated by Capsid Protein VI
Source: PLoS Pathog. 2012 Feb 23;8(2):e1002549. doi: 10.1371/journal.ppat.1002549 (PMC3303589; doi:10.1371/journal.ppat.1002549)

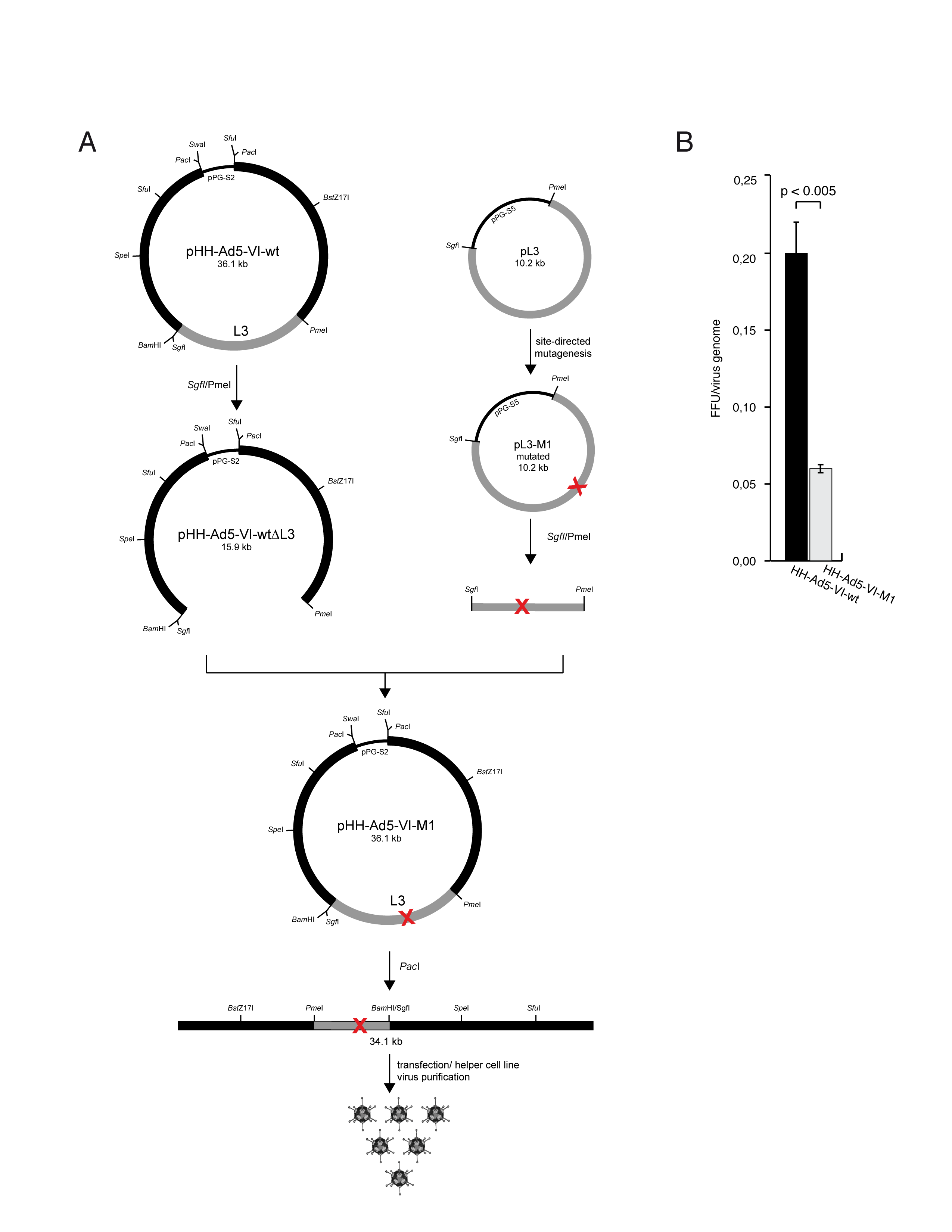

Supplement: Figure S1 — Construction of virus mutant HH-Ad5-VI-M1 by site directed mutagenesis. (A) For the construction of the replication competent virus mutant HH-Ad5-VI-M1, the Ad5 wild type genome in HH-Ad5-VI-wt [H5pg4100; 67] was inserted into the PacI site of the bacterial cloning vector pPG-S2 [67]. It lacks nucleotides (nt) 28593 to 30471 (encompassing most of E3) and contains an additional unique endonuclease restriction site at nt 30955 (BstBI) (nucleotide numbering is according to the published Ad5 sequence from GenBank, accession no. AY339865). In vitro mutagenesis was used to introduce the M1 mutation into the transfer vector pL3 containing the protein VI gene. The resulting transfer vector pL3-M1 was used to replace the SgfI-PmeI fragment in the genome encoding plasmid to generate HH-Ad5-VI-M1. For generation of the HH-Ad5-VI-M1 and the wt control virus, infectious viral DNA was released from the recombinant plasmids by PacI digestion and transfected into the complementing cell line 2E2 [73]. Viral progeny was amplified in 2E2 cells followed by purification on CsCl2 gradients. The integrity of the recombinant virus was verified by restriction digest and DNA sequencing of the entire protein VI gene from isolated viral DNA. (B) For subsequent infection experiments, virus stocks were titered on HEK293 cells [74] and fluorescent forming units were determined by E2A stain for viral replication centers. Virus growth was determined by harvest of infected cells at 24, 48 and 72 h p.i. followed by three freeze/thaw cycles. The cell lysates were serially diluted and virus yield was determined by quantitative E2A stain, 24 h after infection of HEK293 cells as described previously [75]. Viral supernatants were normalized for infectious units (e.g. 50 fluorescence forming units, FFU) prior to use in experiments showing roughly four fold higher ratio of infectious to non infectious particles for the HH-Ad5-VI-wt compared to HH-Ad5-VI-M1. (TIF) [file ppat.1002549.s001.tif]

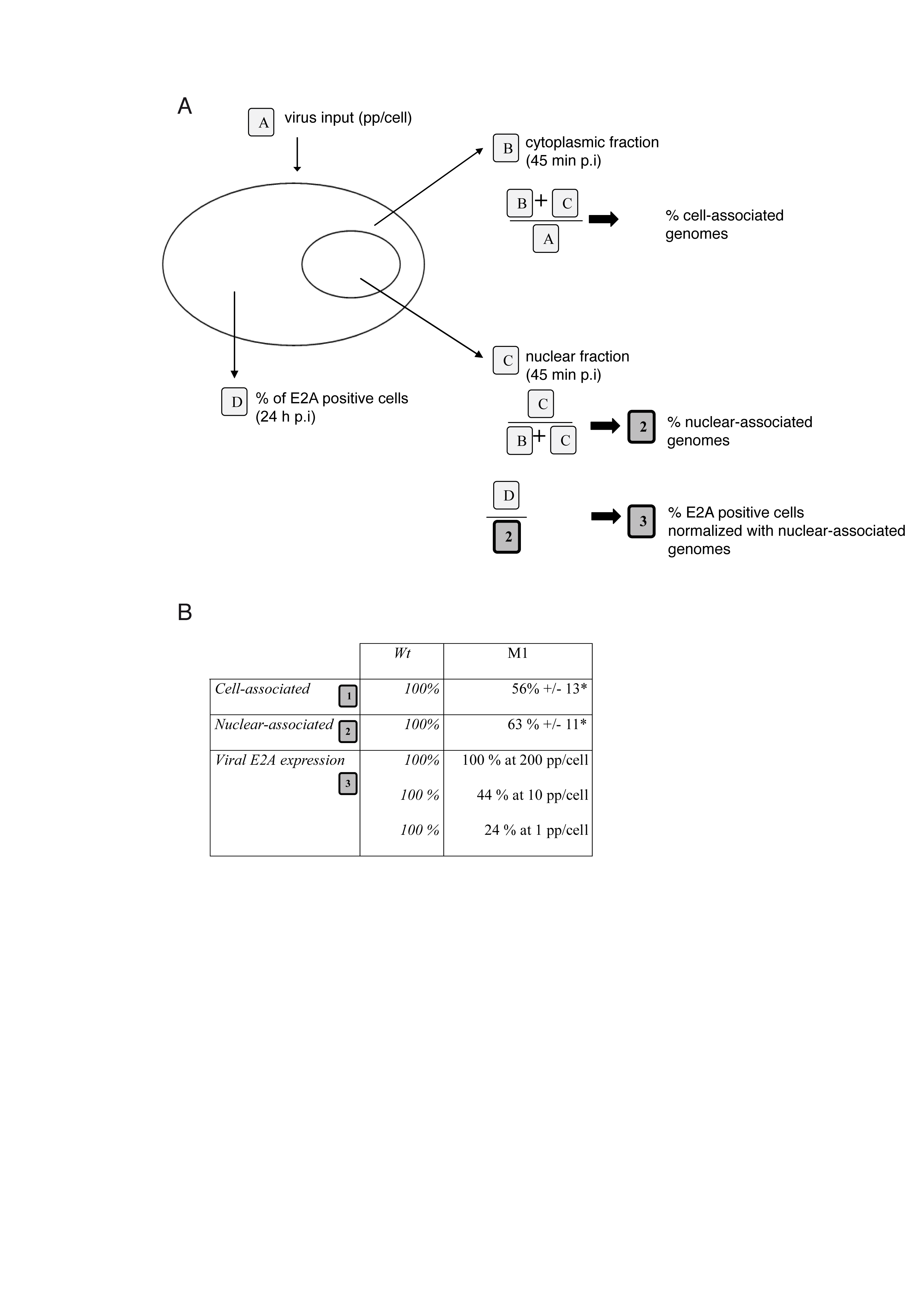

Supplement: Figure S2 — Quantification of viral genomes in fractionated cells. (A) U2OS cells were synchronously infected with replication competent HH-Ad5-wt or HH-Ad5-M1 virus at 200, 10 or 1 physical particles per cell (pp/cell) as virus input –A-. Forty five min after infection, the cytoplasmic –B- and nuclear –C- fractions were separated using nucleo-cytoplasmic fractionation according to the manufacturer's protocol (Pierce). Each fraction was subjected to extraction of the adenoviral genomes using the high pure viral nucleic extraction kit (Roche) according to the manufacturer's instructions. The viral genomes were quantified in –A-, -B- and –C- by qPCR using AQ1 and AQ2 oligonlucleotides to amplify part of the hexon gene [described in 70]. Serial dilutions of a pcDNA3.1 plasmid coding for the Ad5 hexon were used to obtain the standard curve for quantification. The copy number of viral genomes of each fraction was calculated from the Ct-values obtained for each sample. Values were used to determine the cell-associated viral genomes and expressed as percentage of virus input (1 = 100×(B+C/A) reflecting cell binding and virus entry capacity. The percentage of nuclear-associated genomes was calculated and expressed as percentage of total cell-associated genomes (2 = 100×(C/B+C) This value represents viral genomes associated with the nuclear fraction after transport towards the nucleus. To identify how many genomes initiate replication, cells were infected in parallel and stained for replication centers at 24 h p.i. using E2A specific Ab. E2A positive cells were counted and the percentage of E2A positive cells was calculated –D-. In order to discriminate between a viral expression defect and a decrease in virus nuclear transport and accumulation that could account for a loss of expression, the percentage of E2A positive cells was normalized with the percentage of nuclear-associated genome (3 = D/2). To quantify the M1 defects, both the cell-and nuclear-associated genomes of the mutant an [file ppat.1002549.s002.tif]

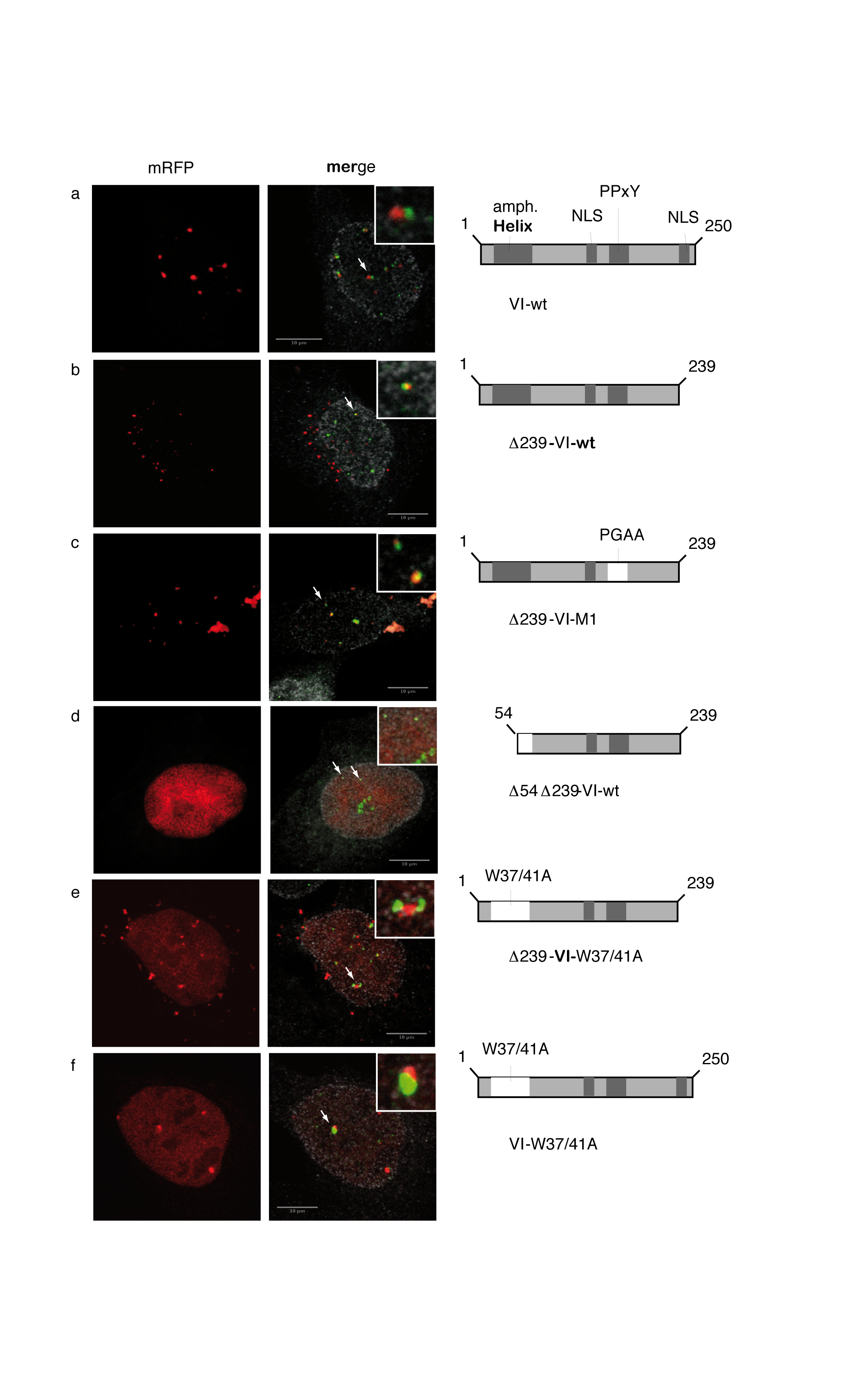

Supplement: Figure S3 — PML-NB association of protein VI requires the amphipathic helix. To identify the domain of protein VI required for PML-NB association, several mRFP tagged constructs for protein VI were transfected into U2OS cells and stained for association with endogenous PML. The mRFP signal is shown in the left column. An overlay of the mRFP protein VI signal (red), endogenous PML (green) and the nuclear envelope stained with MAb 414 (Abcam) against the nuclear pore complex (grey) is shown in the right column. Association of protein VI and PML is depicted by a white arrow and magnified in the top right corner as inset to each overlay panel. Transfected constructs with the functional domains amphipathic helix, nuclear localization signal (NLS) and PPxY motif and their respective modification are indicated to the left. Top to bottom : full length wt protein VI as used in the transfections in Figure 2B (a), C-terminal processed wt protein VI (b), as b with mutated PPxY motif (c), processed protein VI with deleted amphipathic helix (delta 54, d), processed protein VI with two essential tryptophan residues mutated [W37/41]; [ 31] in the amphipathic helix (e) and the same construct as full length version (f). This analysis confirmed that protein VI is targeted to PML-NBs and localized in close proximity to PML. Association of protein VI with PML-NBs was not affected when the PPxY motif was mutated (c) or when processed protein VI, as it is present in the entering virus, was used (b). However, mutating or deleting the amphipathic helix of protein VI changes its distribution from a dot-like pattern towards a partial or complete diffuse, predominant nuclear localization and with loss of its PML-NBs association (d, e, f). These data indicate that the amphipathic helix was a major determinant in targeting protein VI towards PML-NBs. Note that clustering of endogenous PML-NBs in the transfected cells still occurs when the amphipathic helix is mutated or deleted. (TIF) [file ppat.1002549.s003.tif]

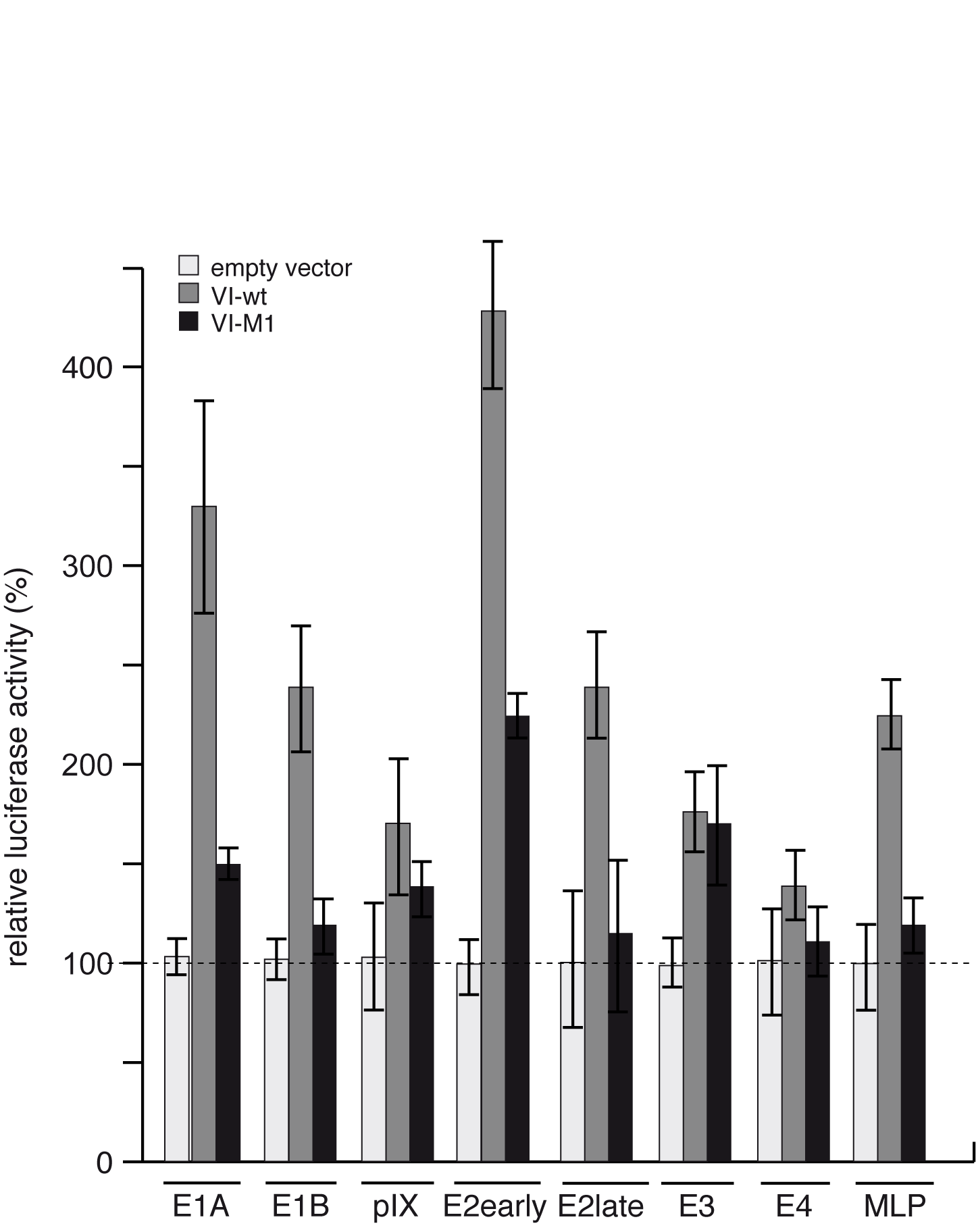

Supplement: Figure S4 — Protein VI mediates adenovirus transcriptional activation of all Ad promoters. Subconfluent H1299 cells were transfected with luciferase reporter plasmids encoding for the E1A-, E1B-, pIX-, E2early-, E2late-, E3-, E4-promoters and the major late promoter (MLP) and effector plasmids expressing VI-wt, VI-M1. Fortyeight hours after transfection, samples were lysed and absolute luciferase activity was measured as described by the manufacturer (dual luciferase kit/Promega). The luciferase activity of each individual promoter was normalized to 100%. The means are presented for three independent experiments. Error bars represent STD. The results show that protein VI stimulates all adenoviral promoters between ∼1.5 to ∼4 fold independent of any other adenoviral protein. Most stimulation is achieved by protein VI-wt compared to protein VI-M1. This is an indication that Daxx repressive mechanisms largely control adenoviral gene expression and that protein VI is an important transactivator that requires the PPxY motif to be fully active. (TIF) [file ppat.1002549.s004.tif]

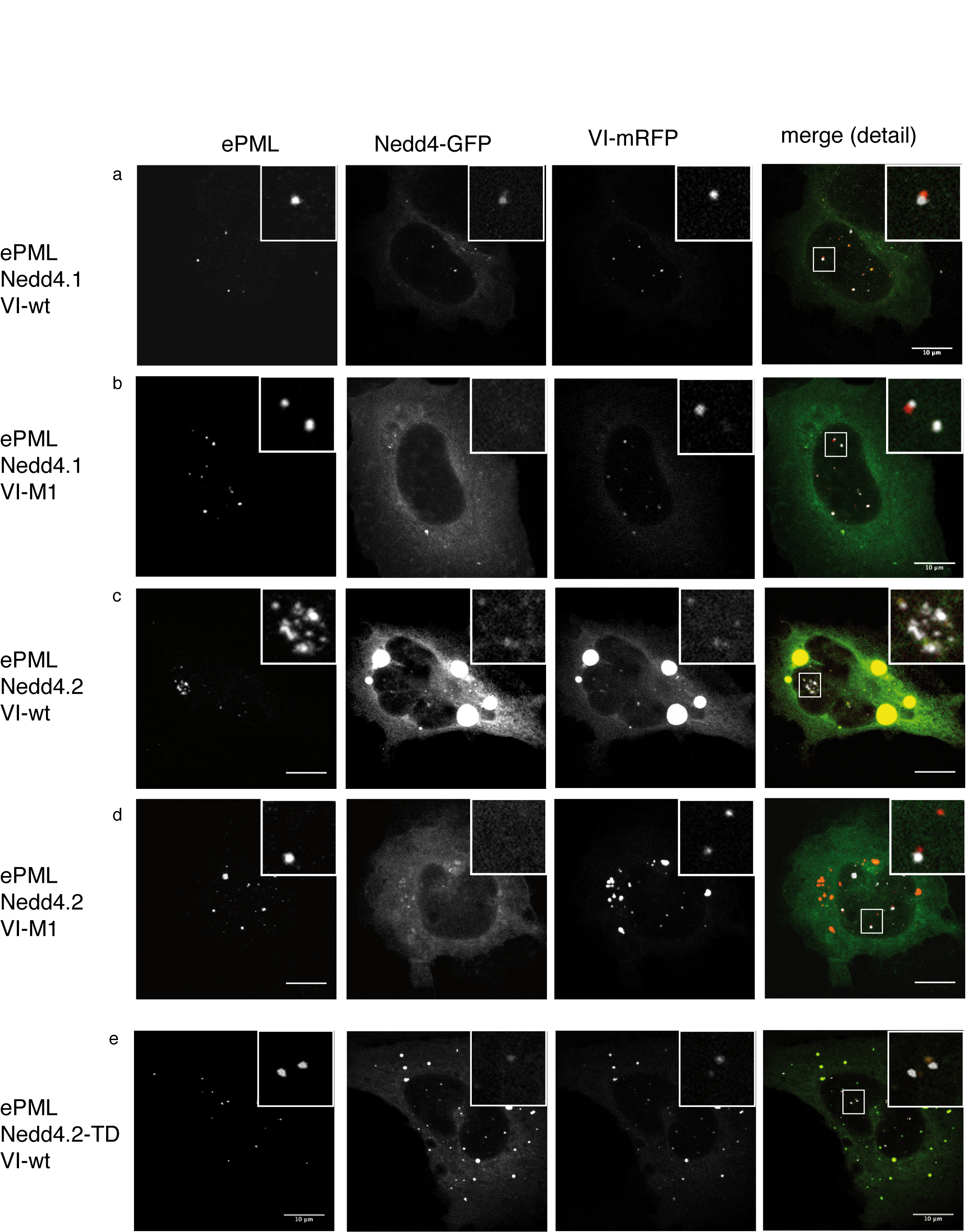

Supplement: Figure S5 — Protein VI targets Nedd4 ligases to PML-NBs via the PPxY motif. U2OS cells were transfected with expression constructs for GFP-tagged Nedd4 ligases and RFP-tagged expression constructs for protein VI-wt or VI-M1 and stained for endogenous PML, as indicted to the left of each row. From top to bottom; Nedd4.1-GFP was cotransfected with VI-wt (a) or VI-M1 (b), Nedd4.2 was cotransfected with VI-wt (c) or VI-M1 (d) or VI-wt was cotransfected with a catalytical inactive mutant of Nedd4.2 (TD, e). An overlay of endogenous PML (grey, first column), Nedd4 (green, second column) and VI (red, third column) is shown in the fourth column. The small inset in each panel shows a magnification of the grey box in the overlay, highlighting colocalization of the three proteins at PML-NBs. Please note that only VI-wt, but not VI-M1, targets Nedd4 ubiquitin ligases to PML-NBs irrespective of the ligase activity. This analysis shows that Nedd4 ligases can be efficiently imported into the nucleus and targeted to PML-NBs, by binding to the PPxY motif of protein VI. We were unable to show that during Ad entry Nedd4 is also translocated into the nucleus or towards PML-NBs due to the bad quality of existing Nedd4 Ab. The observation that transfected protein VI can translocate transfected Nedd4 into the nucleus raises the possibility that incoming particles could also relocate Nedd4 ligases towards the nucleus through association with capsid associated protein VI and alter physiological functions and/or exploit Nedd4 family members to initiate and promote viral replication. (TIF) [file ppat.1002549.s005.tif]

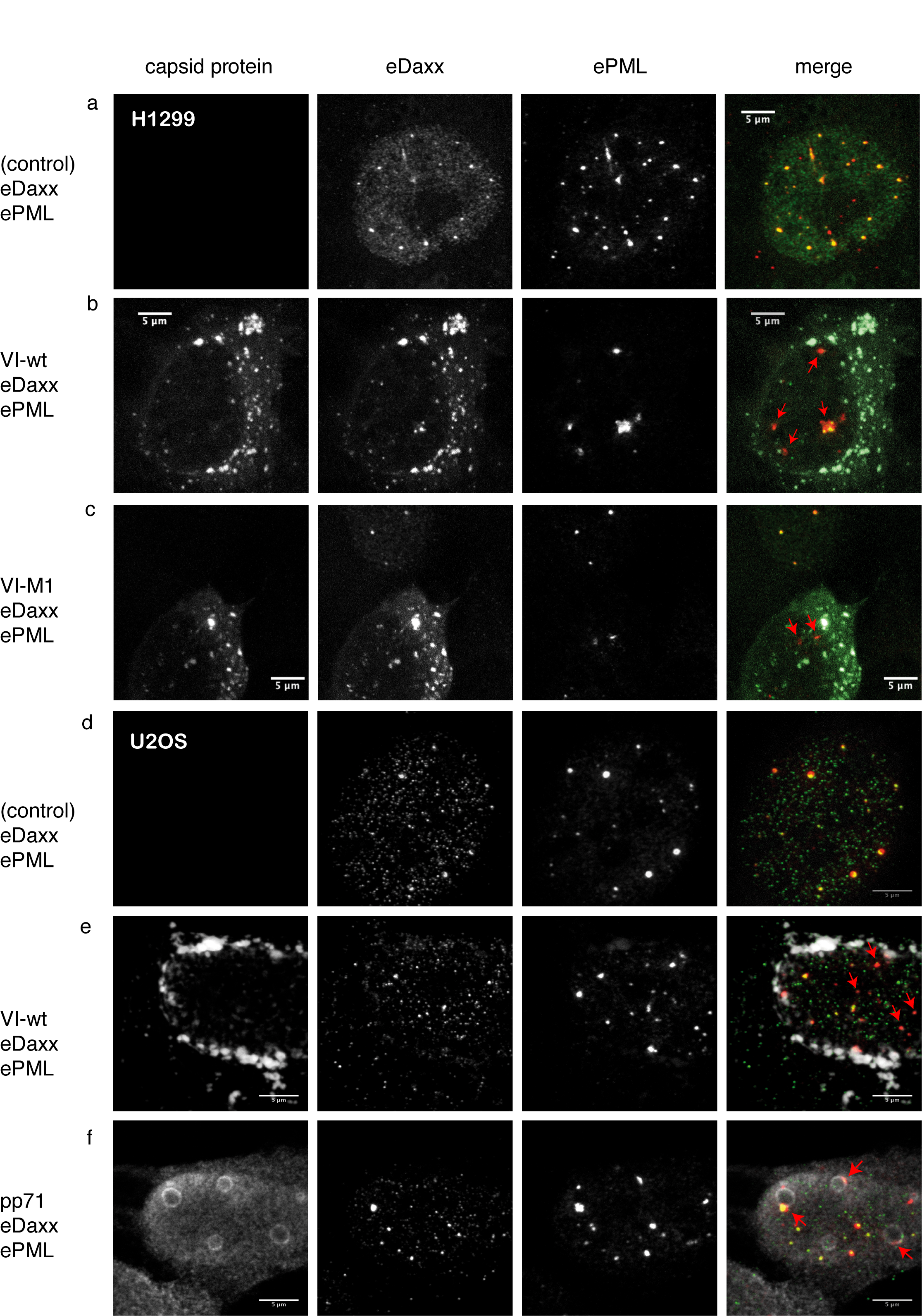

Supplement: Figure S6 — Transfected viral capsid proteins partially displace Daxx from PML bodies. H1299 (a–c) and U2OS (d–f) cells were transfected with either empty control plasmid (a, d) or mRFP-tagged VI-wt (b, e) or mRFP-tagged VI-M1 (c) or with an HA-tagged expression vector for the pp71 tegument protein of the human cytomegalovirus (f, all first columns). Transfected cells were stained for endogenous Daxx (second column) and endogenous PML (third column). The localization of capsid proteins was determined using the RFP signal for VI-wt and VI-M1 or using Ab against the HA-tag to detect pp71 tegument protein. An overlay of all three signals is shown in the last column were capsid proteins are shown in white, Daxx in green and PML in red. Note that red arrows point at PML without (b, c) or with reduced (d) Daxx colocalization or at pp71 induced nuclear structures recruiting Daxx and PML (f). This analysis shows that protein VI (wt and M1) alone is capable of displacing Daxx from PML-NBs and support that protein VI is responsible for the observations made in Figure 6, which show that adenovirus infection results in displacement of Daxx from PML-NBs prior to gene expression. Using different cell lines further supports that protein VI mediated displacement of Daxx from PML-NBs is a genuin property of protein VI. We observed that PML-NB displacement and cytoplasmic accumulation of Daxx was most efficient in H1299 cells while in U2OS cells Daxx was less prominently associated with PML-NBs at steady-state and also less prominently translocated to the cytoplasm upon VI-wt expression. In contrast, expression of VI-M1 let to very efficient Daxx translocation and cytoplasmic colocalization in all three cell lines tested. The observed clustering of PML following transfection is reminiscent of the induced mobility and fusion observed for transfected PML after Daxx displacement in cells microinjected with protein VI as it is shown in Figure 7 and Videos S1 and S2. (TIF) [file ppat.1002549.s006.tif]

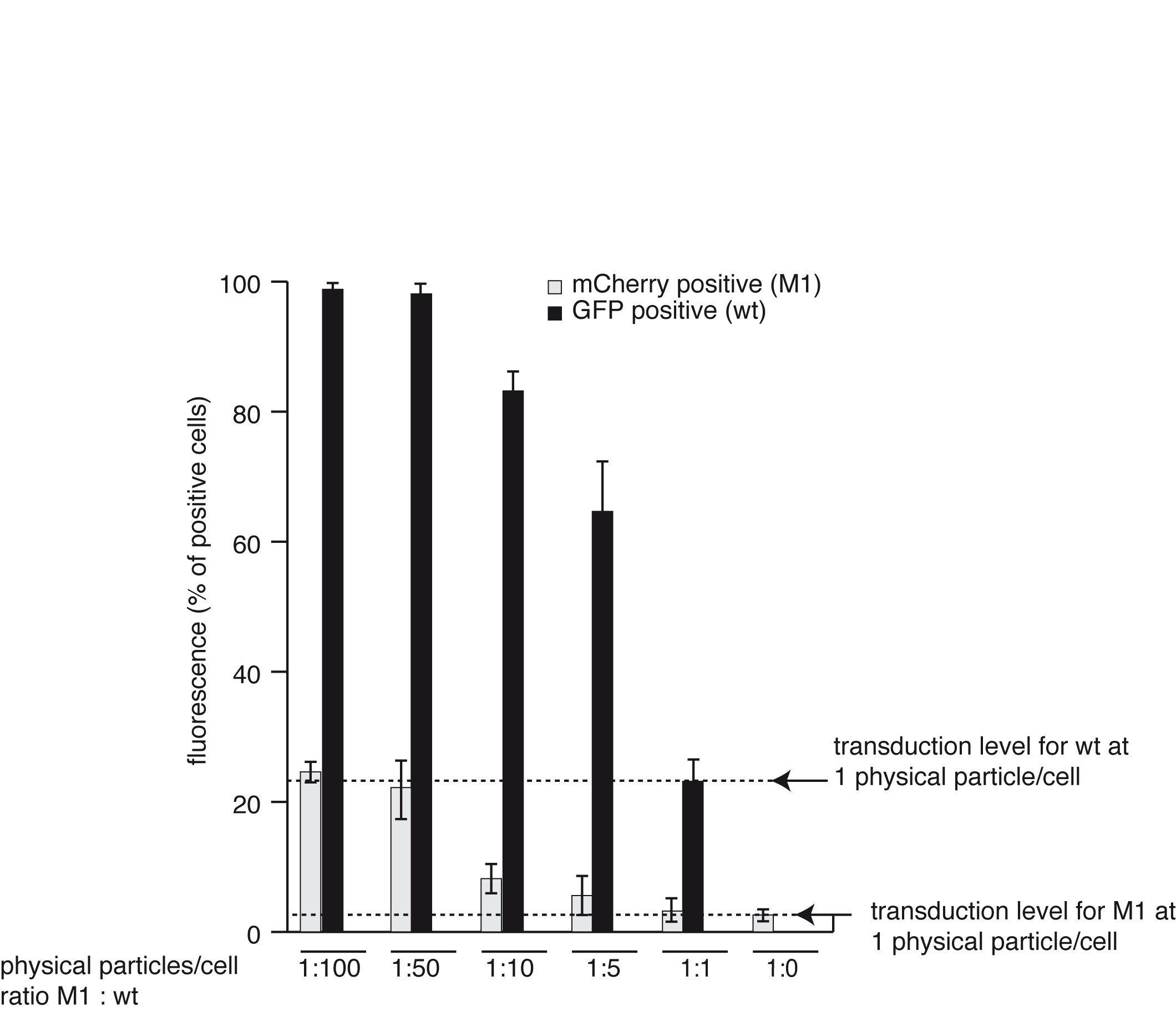

Supplement: Figure S7 — Protein VI-wt activates the CMV promoter of E1-deleted Ad vector particles with M1 mutated protein VI. U2OS cells were transduced with 1 physical particle per cell (pp/cell) of E1-deleted viral vector BxAd5-VI-M1-mCherry (expressing mCherry under CMV promoter control and M1 mutated protein VI) and different amounts of viral vector BxAd5-VI-wt-GFP (expressing GFP under CMV promoter control and wt protein VI). The ratios of M1- to wt-virus are indicated on the x-axes (values in pp/cell). Transduction levels were determined by FACS and are shown separately for M1 (mCherry, light grey bars) and wt (GFP, dark grey bars). The dotted lines indicate wt transduction levels at 1 pp/cell or M1 transduction levels at 1 pp/cell as indicated to the right of the graphic. The increased transduction levels with the M1-virus co-incided with co-transduction of wt-vectors (data not shown). The data show that expression of (CMV-promoter-driven) mCherry from the genome of the E1-deleted Ad vector that encodes protein VI with the M1 mutation is restored when the same cell is also transduced with wt-vector particles that contain protein VI-wt. This observation supports a role for protein VI in activating the CMV promoter and shows that an adenoviral protein (capsid protein VI) can activate the early promoter of a non-related DNA virus (immediate early promoter of HCMV). Because Daxx represses the CMV promoter [45], transactivation by protein VI presumably occurs through removal of Daxx repression. (TIF) [file ppat.1002549.s007.tif]
