# Supplementary material for: Transcriptional Activation of the Adenoviral Genome Is Mediated by Capsid Protein VI
Source: PLoS Pathog. 2012 Feb 23;8(2):e1002549. doi: 10.1371/journal.ppat.1002549 (PMC3303589; doi:10.1371/journal.ppat.1002549)
Supplement: Text S1 — The supporting information contains a list of all antibodies used in this study (Protocol S1), a detailed protocol for the co-immunoprecipitation assays (Protocol S2) and additional references used in Figures S1, S2, S3, S4, S5, S6, S7. (DOC) [file ppat.1002549.s008.doc]

**Text S1- Supporting information**

**Protocol S1. Antibodies.**

Primary antibodies (Ab) against viral factors used in this study included E1A mouse monoclonal Ab (MAb) M73 [1], E1B-55K mouse MAb 2A6 [2], E2A-72K mouse MAb B6-8 [3], E4orf6 mouse MAb RSA3 [4], HCMV pp71 MAb 10G11 [5], L4-100K rat MAb 6B-10 [6], E4orf6 rabbit polyclonal Ab 1807 [7], rabbit polyclonal serum against protein VI [8] and Ad5 rabbit polyclonal serum L133 [9]. Primary Ab against cellular proteins included Daxx rabbit polyclonal Ab (pAb) (Upstate/Millipore), Daxx-01 MAb (Abcam) and Daxx MAb ALX-804-404-C100 (Enzo Life Science), PML rabbit PAb H-238 and PML MAb PG-M3 (Santa Cruz), RFP mouse MAb (Abcam) and ß-actin mouse MAb AC-15 (Sigma-Aldrich, Inc.). Secondary Ab conjugated to horseradish peroxidase (HRP) were from Jackson/Dianova. Secondary Ab used in immunofluorescence were from Jackson/Dianova (Alexa-488, -546) or Sigma Aldrich (Atto-647).

**Protocol S2. Protein Immunoprecipitation Assay (IP).**

## For protein analysis cells were resuspended in RIPA buffer (50mM Tris-HCl/pH 8.0, 150 mM NaCl, 5 mM EDTA, 1 mM DTT, 0.1 % SDS, 1 % NP-40, 0.1 % Triton X-100, 0.5 % sodium deoxycholate) containing 1 % (v/v) PMSF, 0.1 % (v/v) aprotinin, 1 µg/ml leupeptin, 1 µg/ml pep­sta­tin, 1 mM DTT, 25 mM iodacetamide and 25 mM N-ethylmaleimide. After 1 h on ice, the lysates were sonicated and the insoluble debris were pelleted at 15.000 x g/4°C. For IP, protein A-sepharose (3 mg/IP) was coupled with primary specific Ab or unspecific IgG control Ab for 2 h at 4°C and washed twice in RIPA lysis buffer. The Ab bound to protein A-sepharose were added to Pansorbin-Sepharose (50 μl per lysat; Calbiochem) precleared extracts and rotated over night at 4°C. Proteins bound to the Ab-coupled protein A-sepharose were precipitated by centrifugation, washed three times, boiled for 3 min at 99°C in 2x Laemmli buffer and analyzed by IB. For IB, equal amounts of total protein were separated by SDS-polyacrylamide gel electrophoresis (SDS-PAGE) and transferred to nitrocellulose membranes (Schleicher & Schüll/Whatman). Membranes were incubated as described previously [9]. Bands were visualized by enhanced chemiluminescence as recommended by the manufacturer (Pierce) on X-ray films (CEA RP new, medical X-ray film). Autoradiograms were scanned and cropped using Adobe Photoshop CS4 and figures were prepared using Adobe Illustrator CS4 software. Please note that controls are not shown in the figures.

**Supplemental references**

1. Harlow E, Franza BR, Jr., Schley C (1985) Monoclonal antibodies specific for adenovirus early region 1A proteins: extensive heterogeneity in early region 1A products. J Virol 55: 533-546.

2. Sarnow P, Sullivan CA, Levine AJ (1982) A monoclonal antibody detecting the adenovirus type 5-E1b-58Kd tumor antigen: characterization of the E1b-58Kd tumor antigen in adenovirus-infected and -transformed cells. Virology 120: 510-517.

3. Townson SM, Dobrzycka KM, Lee AV, Air M, Deng W, et al. (2003) SAFB2, a New Scaffold Attachment Factor Homolog and Estrogen Receptor Corepressor. J Biol Chem 278: 20059-20068.

4. Marton MJ, Baim SB, Ornelles DA, Shenk T (1990) The adenovirus E4 17-kilodalton protein complexes with the cellular transcription factor E2F, altering its DNA-binding properties and stimulating E1A-independent accumulation of E2 mRNA. J Virol 64: 2345-2359.

5. Kalejta RF, Bechtel JT, Shenk T (2003) Human cytomegalovirus pp71 stimulates cell cycle progression by inducing the proteasome-dependent degradation of the retinoblastoma family of tumor suppressors. Mol Cell Biol 23: 1885-1895.

6. Kzhyshkowska J, Kremmer E, Hofmann M, Wolf H, Dobner T (2004) Protein arginine methylation during lytic adenovirus infection. Biochem J 383: 259-265.

7. Boivin D, Morrison MR, Marcellus RC, Querido E, Branton PE (1999) Analysis of synthesis, stability, phosphorylation, and interacting polypeptides of the 34-kilodalton product of open reading frame 6 of the early region 4 protein of human adenovirus type 5. J Virol 73: 1245-1253.

8. Wodrich H, Henaff D, Jammart B, Segura-Morales C, Seelmeir S, et al. (2010) A capsid-encoded PPxY-motif facilitates adenovirus entry. PLoS Pathog 6: e1000808.

9. Kindsmuller K, Schreiner S, Leinenkugel F, Groitl P, Kremmer E, et al. (2009) A 49-kilodalton isoform of the adenovirus type 5 early region 1B 55-kilodalton protein is sufficient to support virus replication. J Virol 83: 9045-9056.
